# Quantitative Acetylomics Uncover Acetylation-Mediated Pathway Changes Following Histone Deacetylase Inhibition in Anaplastic Large Cell Lymphoma

**DOI:** 10.3390/cells11152380

**Published:** 2022-08-02

**Authors:** Maša Zrimšek, Hana Kuchaříková, Kristina Draganić, Pavlína Dobrovolná, Verena Heiss Spornberger, Lisa Winkelmayer, Melanie R. Hassler, Gabriela Lochmanová, Zbyněk Zdráhal, Gerda Egger

**Affiliations:** 1 Department of Pathology, Medical University of Vienna, 1090 Vienna, Austria; 2 Comprehensive Cancer Center, Medical University of Vienna, 1090 Vienna, Austria; 3 Central European Institute of Technology, Masaryk University, 625 00 Brno, Czech Republic; 4 National Centre for Biomolecular Research Faculty of Science, Masaryk University, 625 00 Brno, Czech Republic; 5 Department of Urology, Medical University of Vienna, 1090 Vienna, Austria; 6 Ludwig Boltzmann Institute Applied Diagnostics, 1090 Vienna, Austria

**Keywords:** histone deacetylases, histone deacetylase inhibitors, SAHA, vorinostat, MS-275, entinostat, proteomics, acetylomics, anaplastic large cell lymphoma, ALCL

## Abstract

Histone deacetylases (HDACs) target acetylated lysine residues in histone and non-histone proteins. HDACs are implicated in the regulation of genomic stability, cell cycle, cell death and differentiation and thus critically involved in tumorigenesis. Further, HDACs regulate T-cell development and HDAC inhibitors (HDACis) have been approved for clinical use in some T-cell malignancies. Still, the exact targets and mechanisms of HDAC inhibition in cancer are understudied. We isolated tumor cell lines from a transgenic mouse model of anaplastic large cell lymphoma (ALCL), a rare T-cell lymphoma, and abrogated HDAC activity by treatment with the HDACis Vorinostat and Entinostat or Cre-mediated deletion of *Hdac1*. Changes in overall protein expression as well as histone and protein acetylation were measured following *Hdac1* deletion or pharmacological inhibition using label-free liquid chromatography mass spectrometry (LC-MS/MS). We found changes in overall protein abundance and increased acetylation of histones and non-histone proteins, many of which were newly discovered and associated with major metabolic and DNA damage pathways. For non-histone acetylation, we mapped a total of 1204 acetylated peptides corresponding to 603 proteins, including chromatin modifying proteins and transcription factors. Hyperacetylated proteins were involved in processes such as transcription, RNA metabolism and DNA damage repair (DDR). The DDR pathway was majorly affected by hyperacetylation following HDAC inhibition. This included acetylation of H2AX, PARP1 and previously unrecognized acetylation sites in TP53BP1. Our data provide a comprehensive view of the targets of HDAC inhibition in malignant T cells with general applicability and could have translational impact for the treatment of ALCL with HDACis alone or in combination therapies.

## 1. Introduction

Acetylation of multiple lysine residues has an important biological function. Moreover, it has emerged as a putative target for cancer therapy [1]. Acetylation levels of histone tails are maintained by the antagonistic activities of histone acetyltransferases (HATs) and histone deacetylases (HDACs). HDACs remove acetyl groups from protein lysine residues and their action can lead to compaction of chromatin, which is associated with transcriptional repression [2]. Furthermore, HDACs are able to deacetylate various non-histone proteins and consequently influence their stability, localization, interactions or general activity [3]. Eighteen human HDACs exist and have been grouped into four classes based on their homology to yeast HDACs. Class I (HDAC1, 2, 3 and 8), class II (HDAC4, 5, 6, 7, 9 and 10) and class IV (HDAC11) are zinc-dependent amidohydrolases. The sirtuin proteins are part of the class III HDACs, which require nicotinamide adenine dinucleotide (NAD) as a cofactor for their catalytic function.

HDAC enzymes play a key role in the regulation of DNA replication, genomic stability and DNA damage response (DDR) and are deregulated in various tumor types [4,5,6]. Many non-histone proteins targeted by HDACs are tumor-suppressor/oncoproteins and therefore directly involved in tumorigenesis [7,8,9,10]. Altogether, this makes HDACs promising targets for antitumor drugs. Individual HDAC inhibitors (HDACis) have already been approved by the Food and Drug Administration (FDA) for the treatment of different T cell malignancies [11,12,13].

HDACis can induce differentiation, apoptosis, as well as autophagic, reactive oxygen species (ROS)-induced or mitotic cell death, senescence and growth arrest [14,15]. The plethora of mechanisms of HDACi-induced cell death reflects the multitude of HDAC substrates. Malignant cells show higher sensitivity to HDAC inhibition compared to non-malignant cells, but HDACis still cause considerable nonselective toxicity. To overcome this, more selective HDACis are being developed [16]. It has been demonstrated that the alterations of gene expression are similar for different HDACis, but some definite differences have been identified. Different responses caused by HDACis depend at least partially on the type of HDACi, concentration and time of exposure, and most importantly, the cellular context [15]. Although many different studies using omics approaches have been investigating HDAC inhibition recently [17], it is crucial that effects of HDACis are further systematically characterized in cell type or cancer type specific models.

Intriguingly, some reports indicate a tumor-suppressive effect of HDAC1 and HDAC2, especially in T cells, where dosage-dependent deletion of the two HDAC enzymes resulted in induction of lymphomas [18,19]. Furthermore, both genetic deletion and chemical inhibition of HDACs accelerated tumorigenesis in a model of acute promyelocytic leukemia [20]. Finally, HDAC1 and HDAC2 are implicated in T cell development and function [21,22,23]. These findings imply that a well-balanced level of HDACs is essential and that in certain cases HDACi treatment might be contraindicated under specific pathological or premalignant conditions.

Nucleophosmin-Anaplastic Lymphoma Kinase (NPM-ALK) positive Anaplastic Large Cell Lymphoma (ALCL) is an aggressive CD30+ non-Hodgkin lymphoma of T cell origin [24]. It is mostly found in children and adolescents [25]. Development of ALK+ ALCL stems from a characteristic translocation between chromosomes 2 and 5, which fuses the shuttling protein nucleophosmin (NPM1) to the anaplastic lymphoma kinase (ALK) [24]. NPM-ALK is constitutively active, which results in induction of several oncogenic signaling pathways [26,27]. Currently, no HDACi is approved for the treatment of ALCL. Fortunately, ALCL tumors are relatively chemo-sensitive with high response rates [28]. In addition, epigenetic mechanisms provide new directions for research and potential novel therapeutic strategies, which could further improve treatment success.

Here, we used a bottom-up proteomic approach to investigate the consequences of reduction of HDAC activity following the treatment with the HDACis Entinostat and Vorinostat or specific deletion of *Hdac1* in cell lines derived from a transgenic NPM-ALK tumor mouse model. A recently published procedure [29] was used to characterize post-translational modifications (PTMs) of histones. To examine changes in the acetylome of non-histone proteins, immunoprecipitation using acetyl-lysine antibodies was performed with a subsequent mass spectrometry approach. Our data provide novel insights into specific histone and non-histone targets of HDACs and highlight possible oncogenic targets and pathways that are repressed by HDAC function in non-cancerous cells.

## 2. Materials and Methods

### 2.1. Mice

Transgenic mice carrying the human NPM-ALK fusion gene under the T-cell-specific Cd4 enhancer-promoter [30] were crossed with mice carrying loxP sites flanking exon 6 of the *Hdac1* gene [31], to generate NPM-ALK *Hdac1*^fl/fl^ mice. The genetic background of mice was mixed (C57Bl/6xSV/129). Mice were kept under specific pathogen-free conditions at the Center for Biomedical Research, Medical University of Vienna, and the experiments were carried out in agreement with the ethical guidelines of the Medical University of Vienna and after approval by the Austrian Federal Ministry for Science and Research (BMWF; GZ.: 66.009/0304.WF/V/3b/2014).

### 2.2. Establishing, Passaging and Culturing of ALK+ Cell Lines

ALK+ cell lines were established from Cd4 NPM-ALK *Hdac1*^fl/fl^ murine T cell lymphomas, which develop at a median age of 20 weeks. Parts of harvested tumor tissue were dissected into smaller pieces in a tissue culture dish containing culture medium. The next day, the cell suspension without the remaining tumor tissue was transferred to a T-25 cell culture flask. Cell lines were monitored daily until they started building characteristic clusters. Cell lines were kept in a humidified incubator at 37 °C in a 5% CO2 environment. The standard culture medium in all experiments was RPMI 1640 medium from GIBCO containing 10% FCS and 1% penicillin/streptomycin.

### 2.3. Transduction of Primary Cell Line with Inducible Cre

Knockout of *Hdac1* in NPM-ALK+ murine ALCL cell lines was achieved by introducing a tamoxifen-inducible Cre recombinase into NPM-ALK *Hdac1*^fl/fl^ cells via lentiviral transduction using an MSCV CreERT2 PuroR vector (kindly provided by Tyler Jacks, Addgene plasmid # 22776). As a negative control, the same vector but with a non-functional Cre recombinase was used (MSCV CreCutERT2 PuroR), where the recombinase was excised by restriction enzyme digestion.

### 2.4. Virus Production and Lentiviral Transduction

The adherent retrovirus producer cell line Phoenix-ECO (ATCC^®^ CRL-3214™, by ATCC) was cultured in Dulbecco’s Modified Eagle’s Medium containing 10% FCS and 1% penicillin/streptomycin. Cells were transfected with the MSCV CreERT2PuroR vector using Lipofectamine 2000 (Invitrogen, Carlsbad, CA, USA) following the manufacturer’s recommendations. After 48 h, the viral particles were harvested. For this, the supernatant containing the packaged virus was transferred to a 15 mL plastic tube and centrifuged for 12 min at 270 g and 4 °C. The supernatant was then filtered through a 0.2 μm filter (Whatman, Maidstone, UK) and stored at 4 °C until further use. For the transduction, 2 × 10^5^ cells were seeded to a 6-well plate in 1.5 mL standard medium. To this, 2 mL of the produced virus-containing supernatant and 8 μL polybrene solution (2 mg/mL stock, by Sigma-Aldrich, St. Louis, MO, USA) were added, and then the plate was centrifuged for 30 min at 200 g and 15 °C (Sigma Laboratory Centrifuges 4K15C, Osterode am Harz, Germany). After 24 h of incubation, the transduction was repeated by adding another 2 mL of viral supernatant and 8 μL polybrene solution and centrifuging as before. The cells were incubated for another 24 h. Cells were selected by the addition of 1 μg/mL puromycin for three weeks.

### 2.5. Conditional Knockout System—4-OHT

To activate the previously introduced Cre recombinase in the ALCL cell lines, cells were treated with 0.5 µM 4-hydroxytamoxifen (4-OHT). The *Hdac1* knockout was confirmed by genotyping and Western blot analysis. Since upon tamoxifen treatment, which induced Cre expression followed by *Hdac1* deletion, some residual HDAC1 expression was still detectable in the mixed clone (*Hdac1* KO), which was most likely due to incomplete Hdac1 deletion and escape from selection, we generated a single clone that showed a complete lack of HDAC1 protein expression on Western blot analysis (*Hdac1* KO SC) (Supplementary Figure S1A,B). Single clones (*Hdac1* KO SC) were selected by serial dilutions of mixed clones following 4-OHT treatment.

### 2.6. Genotyping of Cell Lines

To confirm a successful knockout following the 4-OHT treatment, all cell lines were genotyped. Genotyping was performed with Promega GoTaq Mastermix according to the manufacturer’s suggestions. Primers used: forward—GTT ACG TCA ATG ACA TCG TCC T; reverse—GGT AGT TCA CAG CAT AGT ACT T. PCR program used: initial denaturing step for 3 min at 94 °C followed by 39 cycles for 1 min at 94 °C, 1 min at 62 °C and 3 min at 72 °C and a final extension step for 3 min at 72 °C. The PCR products were visualized on 2% agarose gels containing 0.01 µL/mL Midori Green (Nippon Genetics Europe #MG04, Düren, Germany).

### 2.7. Inhibitor Treatments

Cell lines were treated with respective IC50 concentrations of Vorinostat (SAHA) (Selleckchem, Houston, TX, USA) and Entinostat (MS-275) (Selleckchem, Houston, TX, USA). Original stocks of inhibitors were obtained by dissolving the inhibitors in DMSO according to the data sheets, all further dilutions were completed in culturing medium to minimize the toxic effects of DMSO. To assess IC50 values for inhibitors, 2.5 × 10^4^ cells were seeded in 100 μL culturing medium containing inhibitor per well. IC50 was determined with 8 series of concentrations in triplicates. Cell plates were incubated at 37 °C for 48 h. The viability of cells was determined by Resazurin viability assay. Generation of the dose–response curves and estimation of IC50 values was performed by using nonlinear regression (curve fit) analysis in GraphPad Prism, version 8.00 software (GraphPad Software, vs. 9, San Diego, CA, USA). For all further experiments, cells were grown to an appropriate density, spun down and resuspended in the culturing medium containing the respective drug. Cells were incubated with the drug o/n (16 h) before they were further processed.

### 2.8. Histone Extract Preparation for LC-MS/MS

Histone extracts were prepared according to published protocol [32]. Protein concentration was measured with Bradford Assay (Bio-Rad, Herkules, CA, USA). For chemical derivatization, aliquots of 12 μg of histone extract were used. Microwave-assisted histone derivatization using trimethylacetic anhydride and trypsin digestion (Sequencing grade modified, Promega Corporation, Madison, WI, USA) were performed according to a previously published procedure [29]. The detailed protocol for histone preparation prior to LC-MS/MS is enclosed in the Supplementary Methods.

### 2.9. Preparation of Non-Chromatin Peptides for Acetylomic Analysis

About 1 × 10^8^ cells per sample were harvested and washed twice in ice cold PBS. Each cell pellet was resuspended in 10 mL of urea lysis buffer consisting of 9 M urea in 20 mM HEPES, pH 8.0 with 45 mM sodium butyrate, and 5 M NaCl was added to a final concentration of 50 mM. Precipitated chromatin was separated from the non-chromatin cell fraction by centrifugation (4000 g for 10 min). The supernatant was carefully transferred to a new tube and frozen at −80 °C for further use. Protein concentrations were determined by Micro BCA™ Protein Assay Kit (Thermo Fisher Scientific, Waltham, MA, USA). The following steps were performed according to PTMScan^®^ Acetyl-Lysine Motif [Ac-K] Kit (Cell Signaling Technology, Danvers, MA, USA) manufacturer’s instructions. Purified non-chromatin peptides were reconstituted in 1.4 mL of PTMScan IAP buffer (Cell Signaling Technology, Danvers, MA, USA), cleared by centrifugation at 10000 g at 4 °C for 5 min and peptide concentration was determined by Micro BCA™ Protein Assay Kit. An aliquot of each sample was taken to prepare non-enriched peptide mixtures at a concentration of 1 μg/μL 1 in 1% formic acid (FA, Merck Millipore, Burlington, MA, USA) for LC-MS/MS analysis. PTMScan^®^ Acetyl-Lysine Motif beads (Cell Signaling Technology, Danvers, MA, USA) were used for acetylated peptides affinity enrichment. Enriched peptides were eluted in two steps, each with 55 µL of 0.15% TFA. A detailed protocol for the preparation of non-chromatin peptides prior to LC-MS/MS is enclosed in the Supplementary Methods.

### 2.10. Purification of Samples Prior to LC-MS/MS Analysis

Prior to LC-MS/MS analysis, both histone and non-chromatin peptide samples were desalted on HyperSep SpinTip C18 (Thermo Fisher Scientific, Waltham, MA, USA) according to the manufacturer’s recommendations. Derivatized histones were diluted with 0.1% TFA before desalting, and sequentially eluted with 0.1% TFA in 50% ACN and 0.1% TFA in 75% ACN. Enriched acetylated peptides were eluted sequentially with 0.1% TFA in 40% ACN. All samples were dried in a vacuum concentrator to remove TFA and reconstituted in 0.1% FA (Honeywell, Charlotte, NC, USA) before LC MS/MS analysis. If not specified, all chemicals were purchased from Sigma-Aldrich, St. Louis, MO, USA.

### 2.11. LC-MS/MS Analysis

The samples of all peptide mixtures—(1) derivatized histone peptides, (2) non-chromatin peptides and (3) enriched acetylated peptides of non-chromatin cell fractions—were analyzed on an Ultimate 3000 RSLCnano liquid chromatograph connected to an Orbitrap Fusion Lumos Tribrid mass spectrometer (Thermo Fisher Scientific, Waltham, MA, USA). The analytical column outlet was directly connected to a Digital PicoView 550 ion source with PicoTip SilicaTip (New Objective, Littleton, MA, USA) or Captive spray (ZDV, Bruker, Billerica, MA, USA) emitter. An Active Background Ion Reduction Device (ESI Source Solutions, Woburn, MA, USA) was installed. Derivatized histones were analyzed according to published protocols [29]. Details related to LC-MS/MS analysis including parameters used for measurement of non-chromatin fractions are enclosed in Supplementary Methods.

### 2.12. Data Analysis

All acquired raw data were searched against the cRAP universal contamination database (based on http://www.thegpm.org/crap/, 112 sequences), an in-house Mus Musculus histone database (v210309, 106 protein sequences, generated from UniProt) and UniProt KB Mouse database (v201007, taxon ID:10090, 21989 sequences) using an in-house Mascot search engine (v2.6.2, Matrix Science, London, UK) through Proteome Discoverer software (v2.2.0.388, Thermo Fisher Scientific, Waltham, MA, USA). Derivatized histone peptide search settings were described in previous work [29] with minor changes. Details including parameters used for the database search of non-chromatin fractions are enclosed in the Supplementary Methods. The relative abundances of histone peptides were evaluated according to previously published methodology [33] using R scripts in the KNIME Analytics Platform. Abundances of identified histone peptide forms in particular samples are summarized in Supplementary Table S1.

Label-free quantification of peptides originating from non-chromatin fractions was carried out in Proteome discoverer software. Only peptides with high confidence, minimal 6 amino acid length and Mascot Ion Score at least 30 were used for further processing. Abundances of acetylated peptides in different conditions were evaluated based on peak areas in extracted ion chromatograms. Abundances of non-enriched proteins from non-chromatin fraction in different conditions were assessed using protein intensities calculated using Proteome discoverer software. Peptide and protein abundance data were log2 transformed, then subjected to median normalization. Only peptides and proteins identified in more than one sample per sample group proceeded to two-group comparison using the LIMMA statistical test for differential expression. Fold change (FC) > 1.5 with *p*-value < 0.05 was considered as significant for both peptide and protein results. Peptide results were connected to corresponding protein data. The KNIME Analytics platform was used for statistical evaluation of data. Abundances of non-enriched proteins from non-chromatin fractions in particular samples are summarized in Supplementary Table S2. Abundances of identified acetylated peptides including those showing qualitative changes in particular samples are summarized in Supplementary Table S3. Peptides were then assigned to respective proteins. Since some proteins share a high degree of homology, certain peptides were mapped to more than one protein. In this case, proteins were checked for their homology and only the first match was considered for further analysis. Since more than one peptide can be mapped to a given protein, we also observed in rare instances that peptides with increased and peptides with decreased acetylation levels were matched to the same protein. Data analysis of both histone and non-chromatin fractions is described in detail in the Supplementary Methods.

The mass spectrometry proteomics data have been deposited to the ProteomeXchange Consortium via the PRIDE [34] partner repository with the dataset identifier PXD030503.

### 2.13. Isolation of Proteins, Isolation of Histones and Western Blotting

For isolation of proteins, cell pellets were dissolved in Hunt buffer (20 mM Tris pH 8, 100 mM NaCl, 1 mM EDTA, 0.5% NP-40, protease inhibitor, Roche), frozen in liquid nitrogen, put at 37 °C to thaw, then frozen again and put on ice until completely thawed. The solution was centrifuged and protein concentration of the supernatant was measured using Bradford. Aliquots containing 20 μg of total protein were used for analysis by SDS–PAGE and Western blot. Samples were diluted with 4 × Laemmli buffer (Bio-Rad, Herkules, CA, USA), heated for 5 min at 95 °C and loaded onto SDS–PAGE (6–15%) gradient gels. Gels were run at 0.02 A/gel and subsequently transferred onto nitrocellulose membranes (wet o/n transfer at 25 V, Bio-Rad, Herkules, CA, USA). Membranes were blocked in blocking solution (5% milk powder, 1% PVP, 0.01% NaN3) in TBS-T (TBS containing 1% Triton-X100, Sigma-Aldrich, St. Louis, MO, USA) for 1 h at RT and incubated with primary antibody o/n at 4 °C. After washing with TBS-T, they were incubated with secondary antibody (1:10,000) for 1 h at RT and washed again with TBS-T. The membranes were incubated with chemiluminescent solution ECL plus (GE Healthcare, Chicago, IL, USA) and signals were detected using the ChemiDoc XRS+ (Bio-Rad, Herkules, CA, USA).

For isolation of histones, cell pellets were dissolved in 1 mL ice cold lysis buffer (10 mM Tris pH 6.5, 50 mM Na_2_S2O_5_, 10 mM MgCl_2_, 1% Triton X-100, 8.6% sucrose, adjusted to pH 6.5; right before use the following inhibitors were added: 2 mM Na_3_VO_4_, 10 mM NaF, 0.2 mM PMSF, 1x Protease inhibitor cocktail Roche, 5 mM Na-Butyrate) and centrifuged. Supernatants were discarded and pellet was washed 3 more times with lysis buffer, followed by a washing step with wash buffer (10 mM Tris pH 7.4, 13 mM Na_3_EDTA, adjusted to pH 7.4). Resulting pellets were resuspended in 100 μL 0.4 N H_2_SO_4_ and incubated on ice for 1 h, followed by centrifugation at 12,000 rpm for 10 min. Supernatants were transferred to new tubes and histones were precipitated with 10× volumes acetone at −20 °C o/n. Histones were collected by centrifugation at 12,000 rpm, pellets were air-dried and resuspended in 20 μL MilliQ water. Aliquots containing 2 μg of histones were used for analysis by SDS–PAGE and Western blot as described above.

The following antibodies were used for protein expression analysis: HDAC1 (kind gift from Christian Seiser, clone 10E2), HDAC2 (kind gift from Christian Seiser, clone 3F3), PARP (Cell signaling #9542T), γH2A.X (Phospho-Histone H2A.X Ser139 D7T2V, Cell Signaling #80312S), Beta Actin (Proteintech #66009-I-Ig), Alpha Tubulin (Proteintech #66031-1-Ig) Acetylated Lysine (Cell Signaling #9814), Acetylated and Phosphorylated H3 (Lys9/Ser10) (Cell Signaling #9711), Acetylated H3 (Lys27) (Cell Signaling #8173), H3 (Cell Signaling #44995), Acetylated H2A (Lys5) (Cell Signaling #2576), Acetylated H2AZ (Lys4/Lys7) (Cell Signaling #75336), H2B (Cell Signaling #12364) and H4 (Cell Signaling #13919). Goat anti rabbit IgG HRP conjugate (JD111036047), and rabbit anti mouse IgG HRP conjugated (JD315035008) antibodies were used as secondary antibodies.

### 2.14. Characterization of Apoptotic Cells (Annexin V x DAPI Staining)

Annexin V Staining Solution: one drop of Annexin V Alexa Fluor 488 reagent (Thermo Fisher, Waltham, MA, USA) in 500 μL 1 × Annexin Binding Buffer (Thermo Fisher, Waltham, MA, USA). DAPI Staining Solution: 1:1000 dilution of DAPI Stock solution (1 μg/mL, Sigma Aldrich, St. Louis, MO, USA) in 1 × Annexin Binding Buffer.

The differences between apoptotic and live cells were assessed by incubating the cells with Annexin V and DAPI solutions. A total of 1 × 10^5^ cells washed with 1 × PBS were used. The cell pellet was resuspended in 100 μL of Annexin V staining solution and incubated in the dark for 15 min at RT. Further, the mixture was diluted in a 1:5 ratio with DAPI staining solution, and samples were transferred to FACS tubes (Corning, New York, NY, USA). Sorting cell suspension for different cell populations (live, early apoptotic, late apoptotic and necrotic) was performed on a FACSAria II Flow Cytometer (BD Biosciences, Franklin Lakes, NJ, USA) by acquiring 10,000 events in Pacific Blue and FITC channels. The unstained sample was used to identify target populations and set forward (FSC) and side scatter (SSC) parameters. The analysis was completed in triplicates. Results were analyzed with FlowJoTM Software, version vX.0.7, and further evaluated in GraphPad Prism, version 8.00 software (GraphPad Software, vs.9, San Diego, CA, USA), where an unpaired t-test was used to determine the significance of the changes (*p*-value < 0.05).

### 2.15. Irradiation of Cells

For irradiation of cells, a YXLON Maxishot X-ray unit (Yxlon International X-ray GmbH, Hamburg, Germany) was used. Standard dosimetric quality assurance was performed in regular intervals and the dose rate was found to be constant. Alteration of the time of the irradiation thus defined the delivered dose. The YXLON Maxishot was operated at 200 kV with a tube current of 20 mA and a focus size of 5.5 mm. An additional 3 mm Al and 0.5 mm Cu beam filters were used, which resulted in a dose rate of cca. 1 Gy/min. A single irradiation of 1 min 39 s which equaled 2 Gy was given to each cell line in the T75 culture flask.

### 2.16. Immunofluorescence Staining

Cells were washed 2 × with 1 × PBS and counted. Then, 5 × 10^4^ cells in 5–10 µL were seeded per reaction field onto adhesion slides (Marienfeld #0900000). After a pre-extraction step with 0.1% Tween in PBS (PBST), cells were fixed with 2% PFA in PBS for 20 min at RT. Cells were permeabilized with 0.5% Triton in PBS for 10 min at RT. Slides were washed 3 × with PBS. Cells were blocked with 5% BSA in PBST for 1 h at RT. Primary antibodies were added and slides were incubated o/n at 4 °C. The next day, slides were washed 3× with 5% BSA in PBS and incubated with secondary antibodies for 1 h at RT. Slides were washed 3 × with 5% BSA in PBS and 1 × with PBS. DAPI solution was added (0.2 μg/mL DAPI in PBS) and incubated for 10 min at RT. Slides were washed 2× with PBS and coverslips were mounted with DAKO mounting medium (Agilent Technologies, Santa Clara, CA, USA). Slides were dried o/n at 4 °C before imaging. Slides were imaged using an LSM 700 laser scanning confocal microscope (Carl Zeiss). Primary antibodies used: 1:1000 γH2A.X (Phospho-Histone H2A.X Ser139 D7T2V, Cell Signaling #80312S) in blocking solution, 1:200 TP53BP1 (Bethyl #A300-272A) in blocking solution. Secondary antibodies used: 1:2000 Alexa Fluor 546 goat anti mouse (Invitrogen #A-11018) and 1:2000 Alexa Fluor 488 goat anti rabbit (Invitrogen #A-11070) in blocking solution.

### 2.17. String Analysis

Deregulated non-chromatin proteins and non-chromatin proteins with differential acetylation found in MS-275, SAHA and *Hdac1* KO SC samples were submitted to the String online tool (version 11.5) with the following settings: network type—full String network; active interaction sources: text mining, experiments, databases, co-expression, neighborhood, gene fusion, co-occurrence; minimum required interaction score: medium confidence (0.400). Reactome pathways were used for pathway analysis. For MS-275 samples, the String network for proteins with upregulated acetylation was further imported into Cytoscape (Version: 3.9.0) included in Figure 5E.

## 3. Results

### 3.1. Experimental Setup

**Figure 1:**
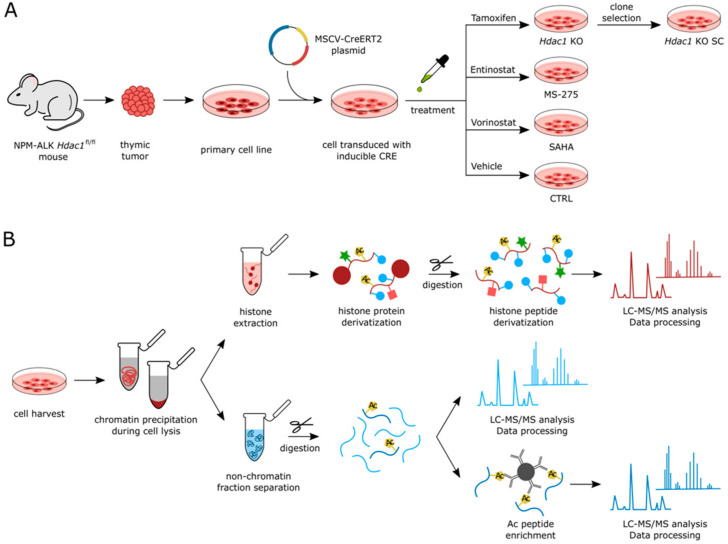
*Experimental design*. (**A**) Generation of mouse cell lines from tumors of transgenic NPM-ALK *Hdac1*^fl/fl^ mice followed by transduction with a tamoxifen-inducible Cre expression plasmid (MSCV-CreERT2). Treatments highlight the experimental groups used in the study; (**B**) sample preparation for bottom-up proteomics. The cells were fractionated into chromatin and non-chromatin fractions. Histones extracted from chromatin were chemically derivatized and digestion with trypsin followed by derivatization of peptide N-termini was performed prior to LC-MS/MS. Aliquots of 10 μL of tryptic peptides obtained from non-chromatin fractions were used for proteome analysis while 3 mg of samples were subjected to enrichment on acetylated peptides prior to LC-MS/MS.

We aimed to investigate the effects of deletion or pharmacological inhibition of HDACs on protein acetylation in ALCL. We employed T cell lymphoma cell lines generated from a transgenic NPM-ALK mouse model harboring *Hdac1* floxed alleles [22,30]. For achieving *in vitro* deletion of *Hdac1*, we additionally introduced a tamoxifen-inducible Cre-recombinase into these cells. The generated NPM-ALK *Hdac1* knockout cells include a mixed clone (*Hdac1* KO), showing some residual HDAC1 protein, and a single clone that displayed a complete lack of HDAC1 protein expression according to Western blot analysis (*Hdac1* KO SC) (Figure 1A, Supplementary Figure S1A,B). Upon *Hdac1* deletion, compensatory HDAC2 upregulation could be observed (Supplementary Figure S1C). In addition to tamoxifen-treated *Hdac1* KO cells, we treated non-induced control cells (CTRL), with two different HDACis. We used the pan-HDACi Vorinostat (SAHA), which inhibits class I and class II HDACs or the class-I-specific HDACi Entinostat (MS-275), which has the highest potency against HDAC1 and significantly lower potency against HDAC2 and HDAC3 [35]. For both compounds, IC50 concentrations were selected (Supplementary Figure S1D). Cells were treated for 16 h (Figure 1A). All samples were prepared in quadruplicates. Changes in the non-chromatin proteome and acetylation levels of histone as well as non-histone proteins were analyzed using a bottom-up proteomics approach (Figure 1B).

### 3.2. Reduction of HDAC Activity Results in Global Changes in the Acetylation Status of Histones

**Figure 2:**
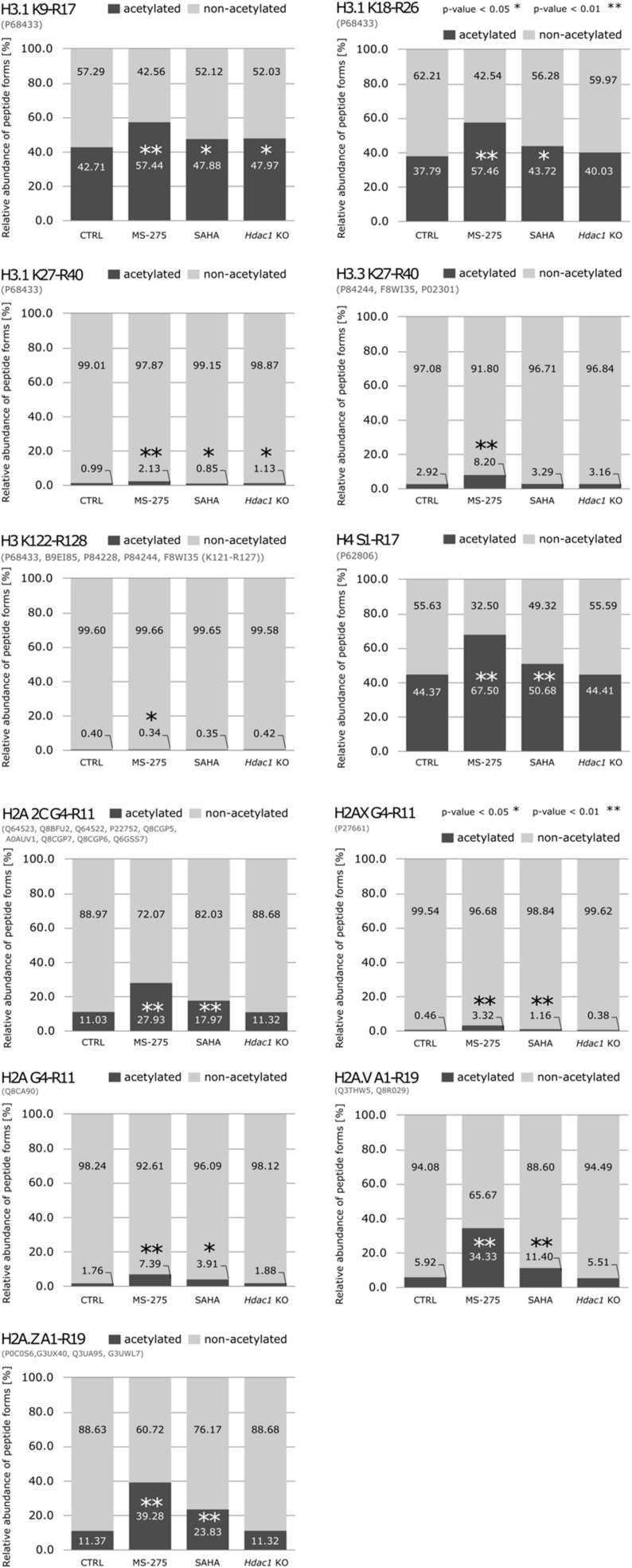
Characterization of global acetylation changes in histone peptides following the reduction in HDAC activity. Impact of deletion or pharmaceutical inhibition of HDACs on global acetylation status of histones. Relative abundance of non- and acetylated peptides represented by geometric mean from biological replicates (N = 4) is shown, and significant differences between treated and control samples are indicated. Given sequence homology between histone variants, certain peptides are shared; peptide sequence identifiers are stated in black font, while accession number(s) for all proteins sharing the same sequence are listed in parentheses below. * *p* < 0.05; ** *p* < 0.01.

First, we quantified changes in histone post-translational modifications (PTMs) caused by deletion or pharmacological inhibition of HDACs in *Hdac1* KO, SAHA and MS-275 treated samples compared to control (CTRL) (Figure 1A,B). Samples were analyzed using a recently published procedure based on histone derivatization with trimethylacetic anhydride prior to LC-MS/MS [29]. We mainly found PTMs localized within lysine-rich histone N-terminal sequences to be significantly affected by HDAC inhibition and to a lesser extent by *Hdac1* deletion (Figure 2 and Supplementary Table S1). The biggest effect on global acetylation status of histones was induced by MS-275 treatment. Acetylation significantly increased by 15% on H3.1K9-R17, by 20% on H3.1K18-R26, by 6% on H3.3K27-R40 and by 23% on H4S1-R17 peptides. Even though the increase in acetylation on H3.1K27-R40 peptide was only 1%, the number of acetylated forms doubled compared to control samples, which would suggest that the majority of H3.1K27-R40 peptides are constitutively non-acetylated. A big increase in acetylated peptide quantity was observed on H2A peptides and involves a 28% increase on H2A.V_A1-R19 and H2A.Z_A1-R19, and 17% on H2A 2C_G4-R11 peptides. SAHA caused milder effects on global acetylation, i.e., an increase by 6% on H2A.V_A1-R19, by 7% on H2A 2C_G4-R11, by 12% on H2A.Z_A1-R11 and by 5% on H4S1-R17 peptides. Further, 5% and 6% increases in acetylated H3.1K9-R17 and H3.1K18-R26 peptides were observed. In general, the effect of genetic deletion of *Hdac1* on histone acetylation was negligible. Only a few peptide forms originating from histone H3 showed significantly increased acetylation levels, while no significant effects were observed on global acetylation profiles of H2A and H4 peptides.

**Figure 3:**
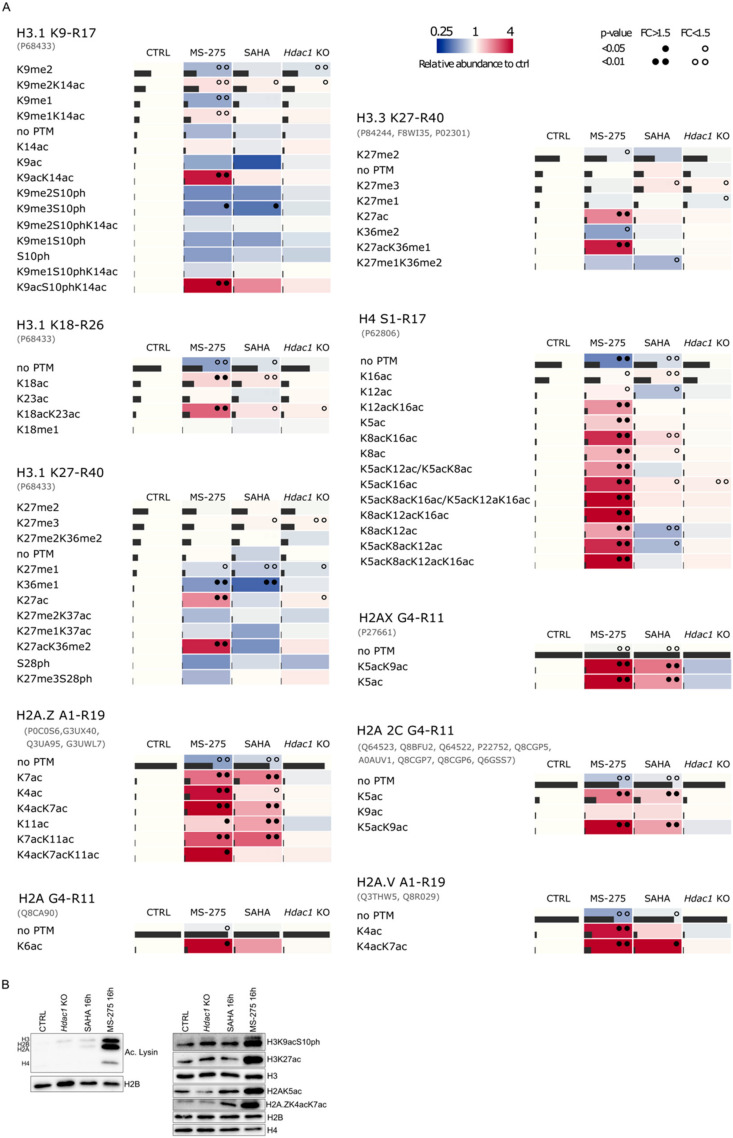
Characterization of acetylation changes in histone peptide forms following the reduction in HDAC activity. (**A**) Graphical arrays depicting the effects of deletion or inhibition of HDACs on the profile of post-translationally modified histone forms. Heatmaps represent the ratios of abundance of peptide forms originating from treated samples to control. Grey column within the heatmap cell reflects relative abundance of a particular peptide form within a respective sequence. Given the sequence homology between histone variants, certain peptides are shared; peptide sequence identifiers are stated in black font, while accession number(s) for all proteins sharing the same sequence are listed in parentheses below. Statistically significant changes in the levels of peptide forms between treated and control cells are indicated by one or two dots (*p* < 0.05/*p* < 0.01). Fold changes FC < 1.5 and FC > 1.5 are depicted by black/empty dots, respectively. Relative abundance of individual forms compared to control is reflected by blue–red color scale. (**B**) Western blot validation of changes in histone acetylation in *Hdac1* KO, SAHA and MS-275 samples. Changes in global acetylation were confirmed using an antibody against acetylated lysine changes in specific post-translationally modified forms were confirmed with respective antibodies against those forms as indicated.

Next, we identified particular post-translationally modified peptide forms associated with changes in global acetylation of histone proteins (Figure 3A and Supplementary Table S1). MS-275 treatment dramatically affected the acetylation state of the H4S1-R17 peptide. A significant increase in all acetylated lysine residues was accompanied by a decrease in the non-modified form. The highest increase was observed for tri- and tetra-acetylated residues (3-fold increase in K5acK8acK12ac, more than 6-fold increase in K8acK12acK16ac and co-eluting K5acK8acK16ac/K5acK12acK16ac forms, and more than 11-fold increase in K5acK8acK12acK16ac). In the case of H3 histone variants, a noticeable increase in the levels of peptides carrying acetylation at positions K9, K14, K18, and K27 was observed. Non-acetylated forms including those with methylation at K9 and phosphorylation at S10 were downregulated. SAHA treatment resulted in smaller changes in H3 and H4 peptides, with fold changes less than 1.5-fold for significant peptides. However, both HDACis strongly influenced the acetylation status of H2A histone variants. Every identified acetylated peptide form of H2A histone variants increased in abundance in MS-275 samples, most of them also in SAHA samples. Changes in modification status caused by *Hdac1* deletion were mild for all histone peptides. A significant decrease was found in the levels of H3.1K9me2, whereas increased acetylation was observed for H3.1K9me2K14ac, H3.1K18ac, H3.1K18acK23ac, H3.1K27ac and H4K5acK16ac. Interestingly, increased levels of peptide forms carrying K27me3 appeared as a common feature of HDAC1 depletion and SAHA treatment, but not following MS-275 exposure. Together, these data suggest that MS-275 treatment induces the highest level of changes in different H2A, H3 and H4 peptides. Effects of genetic *Hdac1* deletion were very mild, which might be due to residual HDAC1 protein or compensatory effects by its homologue HDAC2 (Supplementary Figure S1C). Changes in global acetylation as well as in specific post-translationally modified forms of histone H3 and H2A were validated using Western blot analysis (Figure 3B).

### 3.3. Reduction in HDAC Activity Results in Deregulation of the Non-Chromatin Proteome

**Figure 4:**
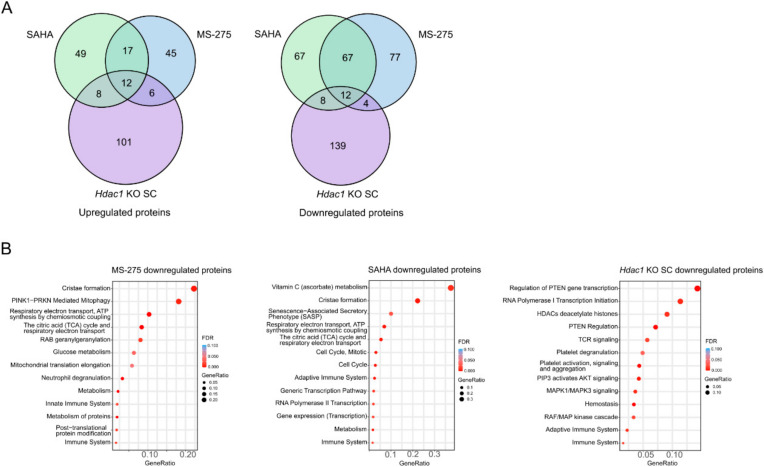
Characterization of the proteome changes following the reduction in HDAC activity. (**A**) Venn diagrams depicting shared up- or downregulated proteins in MS-275, SAHA and *Hdac1* KO SC samples compared to control (LIMMA test, *p*-value < 0.05, FC > 1.5). (**B**) REACTOME pathway analysis of downregulated proteins in MS-275, SAHA and *Hdac1* KO SC samples compared to control (LIMMA test, *p*-value < 0.05, FC > 1.5), using the STRING network analysis tool.

The non-chromatin proteome was analyzed in samples with an *Hdac1* deletion (*Hdac1* KO SC) and samples treated with MS-275 or SAHA using LC-MS/MS and compared to CTRL. In total, 3721 proteins were quantified. In samples treated with HDACis, more proteins were found to be significantly downregulated than upregulated (0.67 > FC > 1.5, *p*-value < 0.05) (Figure 4A). In MS-275 samples, 80 proteins (2.15%) were upregulated and 160 (4.30%) were downregulated. In SAHA samples, similarly, 86 proteins (2.31%) were up- and 154 proteins (4.14%) were downregulated. In the *Hdac1* KO SC sample the response was more balanced with 127 (3.41%) up- and 163 (3.38%) downregulated proteins. HDACs are generally considered to be negative regulators of gene expression, but HDACs were found at active genes as well [36], moreover most studies on HDAC inhibition found roughly the same amount of up- and downregulated genes [17]. While comparing the HDACi treatments with genetic *Hdac1* deletion, we found that the most deregulated proteins were shared between the two HDACi treatments (29 shared up- and 79 shared downregulated proteins) (Figure 4A). In total, 12 upregulated proteins were found to be shared between all three conditions, including heat shock proteins implicated in a wide variety of cellular processes, such as protection of the proteome from stress. Additionally, 12 downregulated proteins were shared between all samples (Figure 4A). They included UBP10 which acts as an essential regulator of p53/TP53 stability. Furthermore, FBXO3 which can activate p53/TP53-dependent transactivation, was downregulated. Lastly, iASPP that inhibits p53/TP53 function was affected. The regulation of the tumor suppressor p53 through acetylation is a known process [37]. Very importantly, HDAC1 and HDAC2 proteins were only found to be deregulated in *Hdac1* KO SC samples, where HDAC1 was expectedly significantly downregulated and HDAC2 was found to be significantly upregulated. HDAC1 and HDAC2 enzymes share a high degree of homology and many functions. Upon *Hdac1* deletion, generally an upregulation of HDAC2 can be observed, or vice versa [38] (Supplementary Figure S1C). Intriguingly, ALK was also among the deregulated proteins and showed upregulated expression in MS-275 samples, while in *Hdac1* KO SC samples it was found to be downregulated. Unfortunately, from the data we obtained it is impossible to determine whether the detected peptides originate from wildtype ALK protein or oncoprotein NPM-ALK resulting from the translocation. Among upregulated proteins ST17B, a positive regulator of apoptosis, could be found in both HDACi-treated samples, while in samples with the *Hdac1* deletion STK3 (pro-apoptotic kinase) and UBE2Z (involved in apoptosis regulation) were upregulated. Proteins involved in apoptosis were found in downregulated proteins as well, namely AIFM1, BAP31 and ASC. In *Hdac1* KO SC samples, PARP2, UBE2T and MLH1—all involved in DNA repair—were upregulated. Proteins involved in DDR were found among downregulated proteins as well, including DPOE4, PAF15 and BRCC3.

All significantly up- and downregulated proteins were separately submitted to REACTOME pathway analysis using the online STRING network analysis tool [39]. Upregulated proteins were not significantly enriched in any pathway. Among downregulated pathways, several metabolic pathways including the “citric acid (TCA) cycle and respiratory electron transport” were affected following the HDACi treatments (Figure 4B). Acetyl-CoA enters the TCA cycle to generate energy; moreover, acetyl-CoA also provides the acetyl groups for acetylation of histone and non-histone proteins [40]. It was shown before that HDACis can disturb the acetyl-CoA equilibrium, which could possibly contribute to their therapeutic efficacy [41]. Additionally, in SAHA samples, “cell cycle” could be observed among downregulated pathways. HDACi are known to induce cell cycle arrest [42,43,44,45]. In the *Hdac1* KO SC sample, downregulated pathways were different as compared to the HDACi treatments and affected oncogenic signaling such as “PTEN Regulation” and “PIP3 activates AKT signaling”. PTEN is an important tumor suppressor that opposes phosphoinositide 3-kinase (PI3K) function, leading to inactivation of AKT and mammalian target of rapamycin (mTOR) signaling [46]. Importantly, the PI3K/AKT pathway is one of the main deregulated oncogenic signaling pathways in ALCL [47]. Likewise, “MAPK1/MAPK3 signaling” and “RAF/MAP kinase cascade”, both part of another relevant pathway in ALCL [48], were affected. Importantly, we detected “HDACs deacetylate histones” among the downregulated pathways.

*Hdac1* deletion or pharmacological inhibition of HDACs resulted in rather moderate perturbations in the non-chromatin proteome, on average only slightly over 6.5% of all quantified proteins were deregulated. However, those proteins are involved in crucial pathways such as cell cycle, DNA repair, apoptosis, the TCA cycle as well as oncogenic signaling pathways. The full list of analyzed proteins can be found in Supplementary Table S2.

### 3.4. Reduction in HDAC Activity Causes Changes in Acetylation Levels of Specific non-Histone Proteins

**Figure 5:**
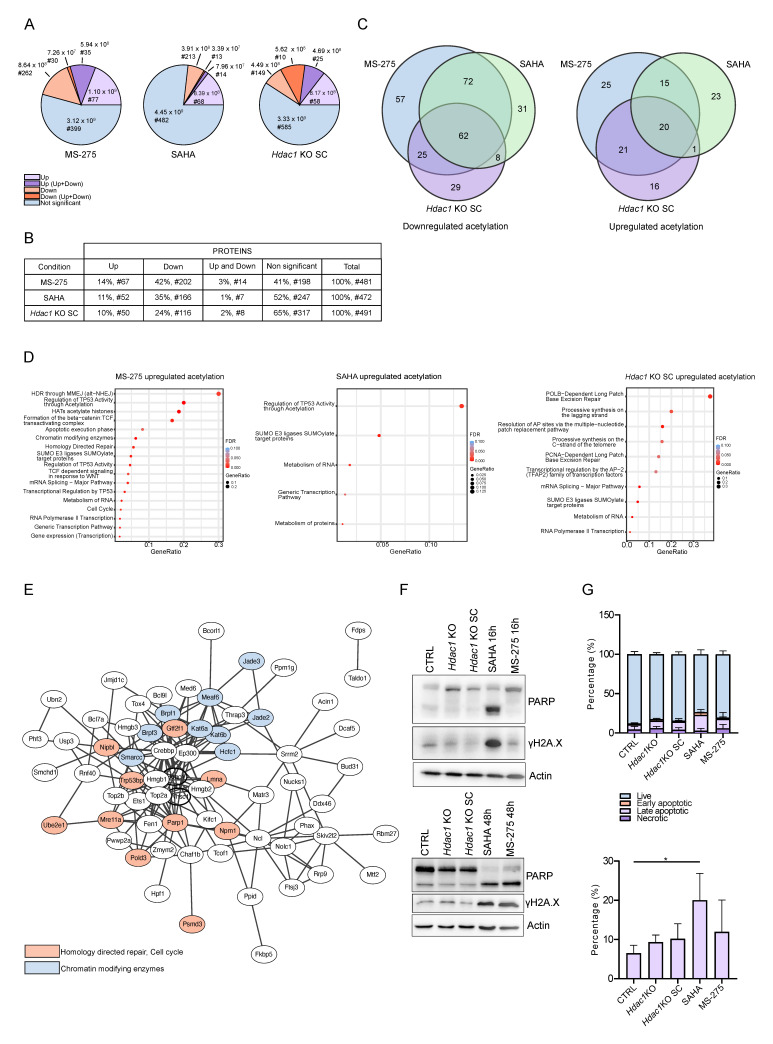
Characterization of acetylation changes in non-histone proteins following the reduction in HDAC activity. (**A**) Pie-charts depicting the proportions of peptides with up- (violet), down- (orange) or non-significant (blue) changes in acetylation in MS-275, SAHA and *Hdac1* KO SC samples compared to control (LIMMA test, *p*-value < 0.05, FC > 1.5). For each category, the number of peptides (#) and the peptide quantity are indicated. For certain proteins, both significantly up- and downregulated acetylated peptides were identified. Those peptides are grouped separately and corresponding up- and downregulated peptides are colored in dark violet and dark orange. (**B**) Number of proteins in individual categories based on identified acetylated peptides in MS-275, SAHA and *Hdac1* KO SC samples compared to control (LIMMA test, *p*-value < 0.05, FC > 1.5). Certain proteins with up- or downregulated peptides included also other peptides with unchanged acetylation levels. (**C**) Venn diagrams depicting shared proteins with up- or downregulated acetylation in MS-275, SAHA and *Hdac1* KO SC samples compared to control (LIMMA test, *p*-value < 0.05, FC > 1.5). Proteins with both up- and downregulated acetylation sites were included in both groups. (**D**) REACTOME pathway analysis of proteins with upregulated acetylation levels in MS-275, SAHA and *Hdac1* KO SC samples compared to control (LIMMA test, *p-*value < 0.05, FC > 1.5), using the STRING network analysis tool. (**E**) The protein network for proteins with upregulated acetylation detected in MS-275 sample compared to control (LIMMA test, *p*-value < 0.05, FC > 1.5), where proteins involved in homology directed repair, cell cycle and chromatin modifiers are highlighted. (**F**) Detection of γH2AX levels and cleavage of PARP by Western blot in CTRL, *Hdac1* KO, *Hdac1* KO SC, SAHA and MS-275 samples, following the 16 and 48 h treatment of SAHA and MS-275 sample with respective IC50 concentrations. (**G**) Characterization of apoptotic cells in CTRL, *Hdac1* KO, *Hdac1* KO SC, SAHA and MS-275 samples. Analyzed in triplicates. Above: fractions of live, early apoptotic, late apoptotic and necrotic cells are shown. Below: fraction of late apoptotic cells is shown. Data are represented as mean ± SD, * *p* < 0.05.

To examine the effect of HDACi treatment or HDAC1 depletion on non-histone protein acetylation in the lymphoma cell lines, we enriched acetylated peptides from the non-chromatin fraction using the PTMScan^®^ Acetyl-Lysine Motif [Ac-K] Kit. Samples with *Hdac1* deletion (*Hdac1* KO SC) and samples treated with the HDACis SAHA and MS-275 were examined using LC-MS/MS analyses after acetylated peptide enrichment and compared to CTRL samples (Figure 1A,B). Altogether, we quantified 1204 acetylated peptides on 603 proteins in enriched samples (across all sample groups). From those, 503 acetylated peptides originating from 346 non-histone proteins showed significant changes in their acetylation levels in samples with *Hdac1* deletion or treated with HDACis compared to control (0.67 > FC > 1.5, *p*-value < 0.05). We observed higher levels of upregulated acetylated peptides compared to downregulated ones in all conditions (Figure 5A). Increased acetylation was related to a relatively low number of highly abundant peptides and corresponding proteins, suggesting selective activity of HDAC enzymes. On the other hand, lower levels of downregulated acetylation were observed, but more different peptides and corresponding proteins were identified (Figure 5A,B, Supplementary Figure S2A). We speculate that downregulated acetylation might be a secondary effect. An exception to this is acetylated peptides with multiple acetylated lysines, where after the inhibition of HDAC activity the peptide pool is shifted towards high acetylation, which means the low acetylation pool of individual acetylation sites is missing and appears downregulated.

Next, we identified shared proteins with significantly altered acetylation levels detected within the three conditions. Proteins with mixed acetylation levels (significant up- and downregulation of acetylation detected) were included in both groups (upregulated and downregulated acetylation) in further analysis. Most shared proteins with increased acetylation were found between the class I HDACi MS-275 and *Hdac1* KO samples (41 proteins) (Figure 5C). Of those, 20 proteins were shared among all three conditions. Since increased acetylation is expected as a primary and direct effect of HDAC inhibition, it seems that in fact the effects of MS-275 inhibition of class I HDACs (with highest potency against HDAC1 [35]) are closer to the effects of *Hdac1* KO as compared to pan-inhibition with SAHA (inhibiting classes I and II HDACs). In contrast, most shared proteins with significantly decreased acetylation levels were found between the two treatments, MS-275 and SAHA, with 134 proteins shared. Remarkably, 62 proteins were shared between all three conditions (Figure 5C). The full list of analyzed proteins can be found in Supplementary Table S3.

### 3.5. Reduction in HDAC Activity Affects Acetylation of non-Histone Proteins Involved in Major Cellular Processes

All proteins with significantly changed acetylation levels were submitted to REACTOME pathway analysis using the online STRING network analysis tool [39]. Proteins with up- and downregulated acetylation sites were analyzed separately, since we assume that only the former is a direct effect of HDAC inhibition. Focusing first on the proteins with increased acetylation, we found similar pathways to be affected in all three conditions. We detected acetylation changes in proteins involved in transcription, such as T2FA (K407ac, K421ac), MTF2 (K23ac) and MED6 (K236ac, K241ac). A multitude of proteins that are part of RNA metabolism were affected including CPSF5 (K23ac), MTREX (K41ac) and DCPS (K9ac, K1ac), with a significant number of proteins involved in splicing, namely PHAX (K323ac), SRRM2 (K2556ac), WBP11 (K13ac), BUD31 (K125ac) and DDX46 (K904ac). Generally, a high proportion of proteins with different acetylation levels upon HDACi treatment or *Hdac1* deletion belonged to protein families associated with chromatin modification. Interestingly, changed acetylation of HDAC1 itself at K412 was observed upon pharmacological HDAC inhibition. Changed acetylation levels were also detected for the acetyltransferases CREBBP (changes on more than 10 different lysines) and EP300 (8 different lysines), as well as KAT6A (K813ac and K816ac) and KAT6B (K380ac, K394ac, K856ac and K860ac)—components of the MOZ/MORF complex, which has a histone H3 acetyltransferase activity. Furthermore, JADE2 (K32ac, K38ac), JADE3 (K32ac, K35ac and K38ac) and PEREGRIN (K382ac)—all subunits of the HBO1 complex, which specifically mediate acetylation of histone H3 at Lysin 14 (H3K14ac), were affected. MEAF6 (K69ac, K74ac and K151ac) also had significantly changed acetylation levels. MEAF6 is a component of the NuA4 histone acetyltransferase complex which is responsible principally for acetylation of histone H4 and H2A. MEAF6 can also be part of MOZ/MORF or HBO1 complexes. BRPF3 (K96ac, K98ac and K322ac), scaffold subunit of MOZ/MORF or HBO1 complexes, was found to be differentially acetylated as well. Changes in acetylation were also detected in HCFC1 (K836ac) that is involved in control of the cell cycle and is able to tether the chromatin modifying SET1/ASH2 histone methyltransferase (HMT) complex and SIN3/HDAC complex. Lastly, acetylation levels of histone methyltransferase NSD2 and SMARCC1 (K353ac), a subunit of the SWI/SNF complex, which is responsible for chromatin remodeling, were affected. Upon SAHA and MS-275 treatment, pathway “regulation of TP53 activity” was affected. In *Hdac1* KO and MS-275-treated samples, hyperacetylated proteins involved in DDR pathways such as base excision repair and homology directed repair were detected, namely PARP1 (K400ac, K418ac, K520ac), TP53BP1 (K1357ac), FEN1(K373ac), MRE11A (K671ac), PSMD3 (K14ac), NIPBL (K775ac), LIG1 (K208ac), PMS2 (K 210ac) and YY1 (K351ac) (Figure 5D). The interconnectedness of the proteins with increased acetylation detected in MS-275 treated samples can be observed in Figure 5E, where chromatin modifiers and proteins involved in homology directed repair and cell cycle are highlighted. It was shown before that HDACi treatment can induce G/M2 cell cycle arrest [42,43,44,45] and that HDACs are involved in DDR [49]. Proteins involved in cell proliferation and DNA replication included POLD3 (K429ac), RPA3 (K33ac), PCNA (K80ac) and NPM1 (K141ac, K227ac) (Figure 5D). Acetylated lysines detected on NPM1 (K141ac and K227ac) do not lie in the oligomerization domain, that is also part of the fusion oncoprotein NPM-ALK, suggesting that only the full length NPM1 protein was affected. Together, these data suggest that known HDAC-dependent pathways including cell cycle regulation and DDR are directly affected by changes in acetylation of respective pathway associated proteins. Interestingly, the significant association of hyperacetylated proteins with chromatin-related pathways suggest a strong cross talk among various chromatin regulating proteins (Figure 5D).

Proteins with downregulated acetylation levels were mostly involved in metabolism in all three conditions. Highlighted pathways included the “metabolism of proteins”, “TCA cycle” and “glucose metabolism”. Effects of HDACi treatment on metabolism have been already documented before [41,50]. Besides changed acetylation levels, proteins involved in the TCA cycle were also found to be deregulated following the HDACi treatments (Figure 4B). It was shown before that HDACis can disturb the acetyl-CoA equilibrium. This could potentially lead to depletion of acetyl-CoA, which could be especially important in mitochondria, since the conditions in mitochondria may favor non-enzymatic lysine modification by acetyl-CoA [51]. In favor of this speculation, approximately a quarter of non-histone proteins with downregulated acetylation that we detected were mitochondrial proteins (Supplementary Table S4). Again, proteins detected in all three conditions seem to play a role in cell cycle and G2/M checkpoints. (Supplementary Figure S2B).

Altogether, we show that HDAC inhibition or specific HDAC1 depletion affects diverse cellular processes, involving most likely direct and indirect alterations of protein acetylation levels. Interestingly, some of the acetylation sites discovered on various proteins were never described before. Based on pathway analysis it seems that the effects of HDAC inhibition in ALCL cells are similar to the effects of HDACi treatments described in the literature for other cancer cell types. Intriguingly, the pathways affected were highly similar for cells with genetic deletion of *Hdac1* and cells treated with HDACis.

### 3.6. Reduction in HDAC Activity Affects Acetylation of DDR Proteins

To further dissect our results on acetylated proteins, we specifically focused on the pathways involved in DDR, since it is known that HDACis induce DNA damage, which can normally be repaired by non-transformed cells [52]. We treated ALCL cell lines with MS-275 and SAHA for 16 h (to match the conditions used for mass spectrometry analysis) and 48 h and compared the protein expression of DNA damage induced proteins in untreated control and Hdac1 KO samples by Western blot analysis. Elevated levels of γH2AX and cleavage of PARP were confirmed following the HDACi treatments (Figure 5F). Interestingly, although both inhibitors were used at IC50 concentrations, a difference in the response kinetics could be observed between MS-275 and SAHA treatments, with upregulation of γH2AX and complete cleavage of PARP already after 16 h of treatment with SAHA, but only after 48 h treatment with MS-275. This is in contrast to the fact that protein acetylation levels after 16 h of MS-275 treatment were much more affected as compared to SAHA treatment. Furthermore, increased levels of apoptotic cells were confirmed in SAHA and MS-275 samples after 48 h treatments using Annexin staining and FACS analysis (Figure 5G). It was demonstrated before that aminobenzamides (MS-275) are inhibitors with slow association and dissociation kinetic rates and hydroxamates (SAHA) have rapid kinetic binding properties [53], which is also in line with a previously observed delayed cellular response on acetylation levels of distinct histone sites [54]. This suggests that earlier time points after SAHA treatment would better enable to fully capture all acetylation changes, whereas for MS-275 treatments later time points might be beneficial. Different to HDACi-treated cells, deletion of Hdac1 resulted in much smaller changes in γH2AX levels compared to control cells (Supplementary Figure S3C) and did not result in significant PARP cleavage (Figure 5F). Of note, PARP1 showed increased acetylation levels following HDACi treatments.

Furthermore, we discovered that the acetylation levels of TP53BP1, a protein crucial for DDR, were significantly increased both after HDACi treatment and *Hdac1* deletion. Intriguingly, TP53BP1 has a central role in the choice of specific double strand break DSB repair pathways, which is dependent on histone acetylation signatures [55]. Acetylation of H4K16 results in decreased TP53BP1 binding to sites of DSBs, thereby directing DDR from non-homologous end joining (NHEJ) towards homologous recombination (HR). In addition, acetylation of H2AX on K5 was shown to impact DDR. Our mass spectrometry analysis also detected increased levels of K5ac and K9ac on H2AX peptides. Phosphorylation of TP53BP1 is critical for modulating TP53BP1 function in DDR, less is known about other PTMs of TP53BP1. Here we show that TP53BP1 can be acetylated on at least two different lysine residues, K1357 and K1664, respectively (Supplementary Figure S3A). Following the treatment with HDACis or genetic KO of *Hdac1*, the acetylation on K1357 significantly increased. Since it is known that acetylation is important in many cellular processes and it was also shown before that several other DNA repair proteins can undergo protein acetylation [56,57], we hypothesized that it could potentially also modulate TP53BP1 function during DDR. The K1357 acetylation site of TP53BP1 lies in the MDC1 interaction domain that is located between residues 1288-1409 of TP53BP1 (Supplementary Figure S3A). MDC1 is another DNA damage mediator required for checkpoint mediated cell cycle arrest in response to DNA damage. It was previously demonstrated that the TP53BP1–MDC1 interaction is required for the recruitment of TP53BP1 to sites of DNA breaks, which is crucial for an efficient activation of the DDR. It is also suggested that the interaction between TP53BP1 and MDC1 plays a role in the regulation of mitosis [58]. Acetylation of this site could therefore influence the aforementioned interaction and further finetune TP53BP1 function in DDR. Furthermore, the amino acid sequence motif around K1357ac is conserved from mouse to human, suggesting an indispensable function throughout evolution (Supplementary Figure S3A).

### 3.7. Acetylation of TP53BP1 Does Not Disrupt the Colocalization of TP53BP1 and γH2AX Foci during DNA Damage

In order to study the functional role of TP53BP1 acetylation for DDR, we treated cells with SAHA or MS-275 for 16 h followed by irradiation with 2 Gy to induce DNA double strand breaks. We also included untreated control cells that underwent irradiation and *Hdac1* KO cells. In control cells, γH2AX foci formation was induced 1.5 h after irradiation, as observed by immunofluorescence staining (Supplementary Figure S3A,B). Pre-treatment with HDACis induced a higher number of γH2AX foci, which also showed increased staining intensity. Co-staining for TP53BP1 revealed a prominent overlap with γH2AX foci following DNA damage in untreated and HDACi pre-treated irradiated samples, suggesting that acetylation of TP53BP1 on K1357 did not affect TP53BP1 recruitment to sites of DNA damage. In addition, we detected a higher number of mitotic cells upon HDACi treatment, with chromosomes with highly enriched γH2AX, which might be due to G2/M phase arrest following treatment and irradiation [42,43,44,45] (Supplementary Figure S3A,B). Co-immunofluorescence for TP53BP1 revealed that γH2AX positive chromosomes were devoid of TP53BP1 staining. This is in line with previous studies, suggesting that DNA damage foci in mitosis are lacking TP53BP1 and that TP53BP1 remains excluded from the chromatin until cells progress into G1 phase [59,60].

Taken together, our data show that HDAC inhibition increased DNA damage in non-irradiated and irradiated cells but did not interfere with TP53BP1 recruitment to sites of damage. Due to the fact that HDAC inhibition affects both histone modifications and the acetylation of DNA repair proteins including PARP1 and TP53BP1, it will be important to dissect the functional consequences on DDR in more detail in the future.

## 4. Discussion

Although many studies use transcriptional changes as a molecular read-out, this does not take into account the enormous complexity of post-transcriptional, translational and post-translational regulatory processes. It is therefore crucial to further study the role of individual PTMs. In this study we elucidated the targets of HDACs in a murine T cell lymphoma model of ALCL by either pharmacological inhibition of HDACs or genetic deletion of *Hdac1*. We showed substantial changes in acetylation levels of histone as well as non-histone proteins in all conditions. Similar to previous studies, we demonstrate that affected acetylated proteins are involved in major cellular processes. Moreover, we uncovered novel acetylation sites that have not been described before.

The major changes in acetylation of proteins following the *Hdac1* deletion were similar to effects induced by the class I specific inhibitor MS-275 or the pan-inhibitor SAHA. We observed bigger changes in acetylation levels in the MS-275 sample compared to the *Hdac1* KO sample. This is probably due to simultaneous inhibition of HDAC1, HDAC2 and HDAC3 by MS-275. In addition, we observed a compensatory upregulation of the HDAC2 enzyme upon the genetic deletion of *Hdac1*. HDAC1 and HDAC2 enzymes share a high degree of homology and many functions in part through their co-occurrence in repressive chromatin complexes. Upon *Hdac1* deletion, HDAC2 was likely able to take over some of the HDAC1 roles and therefore the observed changes were reduced. Further, compared to catalytic inhibition by chemical compounds, the genetic deletion of *Hdac1* causes the complete loss of the HDAC1 protein including its potential scaffolding function [61], which could influence protein acetylation. In order to specifically investigate the contribution of HDAC1 catalytic function, it will be interesting to assess the acetylation changes in cells expressing catalytically inactive HDAC1.

While the changes in the acetylome were comparable between the genetic *Hdac1* deletion and pharmacological inhibition of HDACs, more differences could be observed in the non-chromatin proteome. About 70% of proteins with down- and upregulated acetylation in the *Hdac1* KO SC could also be found in MS-275 treated cells. In contrast, only 14.2% of upregulated proteins and 9.8% of downregulated proteins in *Hdac1* KO SC were simultaneously detected in MS-275 samples. Furthermore, downregulated proteins in HDACis-treated samples were mostly involved in TCA cycle and metabolism, in contrast deregulated proteins in *Hdac1* KO SC samples were part of (oncogenic) signaling processes. Why there is a big difference in deregulated proteins, but not in proteins with changed acetylation levels, between genetic deletion and pharmacological inhibition, remains to be further investigated.

We also observed a difference in the kinetics of response of MS-275 and SAHA treatments, although both inhibitors were used in respective IC50 concentrations. In the case of SAHA treatment, the acetylation changes were smaller, while upregulation of γH2AX and complete cleavage of PARP was seen already after 16 h of treatment. On the other hand, the acetylation changes after 16 h MS-275 treatment were much bigger, but the upregulation of γH2AX and complete cleavage of PARP was only seen after 48 h of treatment. Generally, it was demonstrated before that aminobenzamides (MS-275) showed slower binding kinetics with HDAC-containing protein complexes when compared to hydroxamates (SAHA), which was in line with delayed cellular response on acetylation levels of distinct histone sites [54]. This could mean that with the fast-binding SAHA, we missed the biggest peak of acetylation changes, but we were able to see full effects of the drug on DDR. Conversely, with slow-binding MS-275, we captured a peak of acetylation changes, but would need to wait longer to observe increased DNA damage and apoptosis. Thus, the kinetics of different inhibitors should be considered in future experiments.

We detected many well-known acetylation sites on histone variants of H3 and H4, as well as H2A. We omitted the evaluation of H2B variants in our study as obtained results are difficult to interpret (already described in [29]). In the non-histone protein fraction, besides the increased acetylation, we detected many peptides with downregulated acetylation levels as well. While increased acetylation is a direct effect of HDAC inhibition, we speculate that loss of acetylation might be a secondary effect. In agreement with published results on the impact of HDAC inhibition on apoptosis, cell cycle arrest and changes in metabolic pathways [15,50], deregulation of proteins involved in aforementioned pathways was also detected in our proteome analysis. This could potentially also affect PTMs of proteins involved in cellular metabolism. On the other hand, the hyperacetylation of chromatin and specific nuclear target proteins, might result in intracellularly depleted acetyl-CoA levels and lower acetylation of proteins involved in metabolic processes. This might especially concern non-enzymatic acetylation as described for mitochondrial proteins, which largely depends on the availability of acetyl-CoA pools [51,62].

Many non-histone proteins with changes in acetylation levels were chromatin-associated proteins. We also observed increased acetylation levels of HDAC1 following the HDACi treatment. Remarkably, changed acetylation levels were also detected in acetyltransferases such as CREBBP, EP300, KAT6A and KAT6B as well as different subunits of the HBO1 and MOZ/MORF complexes, which both have acetyltransferase activity. This suggests that HDACs itself and HATs are potential HDAC targets and that there is a crosstalk between these antagonizing enzymes.

Upon treatment, we observed increased DNA damage, which is a known effect of HDACis [49]. We further identified two novel lysine acetylation sites in TP53BP1, an important protein in DDR. TP53BP1 was previously reported as an acetylated protein and acetylation of K1626/1628 in the UDR motif of TP53BP1 disrupted the interaction between TP53BP1 and nucleosomes [63]. One of the newly identified acetylated lysine residues exhibited significant increases in acetylation after inhibitor treatment as well as in *Hdac1* KO samples. Interestingly, the respective site lies in the domain important for interaction of TP53BP1 with MDC1. Although we could not find major differences in TP53BP1 recruitment to γH2AX foci in HDACi-treated cells compared to non-treated irradiated cells, TP53BP1 acetylation might still be essential for the regulation or selection of specific DDR pathways. Overall, DDR is dependent on an intricate interplay of chromatin and DNA damage repair proteins that are all targets for distinct PTMs including acetylation and phosphorylation. In order to dissect the individual functions of these PTMs, it will be essential to perform site-directed mutagenesis to better understand the individual aspects of chromatin and protein modifications for efficient DDR. Furthermore, based on increased DNA damage and changed acetylation levels in DDR proteins (including PARP1) we observed following the HDACi treatments it would be worth testing the synergy between HDACis and PARP inhibitors in this model. The combination of these drugs already proved promising in other types of lymphomas [64,65,66].

Altogether, our data provide evidence that HDAC inhibition or specific HDAC1 depletion affects diverse cellular processes. Importantly, the effects of genetic *Hdac1* deletion mirror pharmacological HDAC inhibition, as evident by common changes in protein acetylation. Based on the observations, that deregulated and hyperacetylated proteins are involved in cell cycle, oncogenic signaling and DNA damage response and that HDACi treatments result in increased DNA damage and apoptosis in ALCL cells, HDAC inhibition could be considered as a possible therapeutic intervention for patients suffering from these malignancies. In conclusion, our quantitative proteomics study provides a rich source of deacetylation targets of HDAC1 and small molecule inhibitors, which is essential to study acetylation dynamics in cancer cells and in general.

## Data Availability

The mass spectrometry proteomics data have been deposited to the ProteomeXchange Consortium via the PRIDE partner repository with the dataset identifier PXD030503. [http://proteomecentral.proteomexchange.org/cgi/GetDataset?ID=PXD030503].

## Supplementary Materials

The following supporting information can be downloaded at: https://www.mdpi.com/article/10.3390/cells11152380/s1, Supplementary Figure S1: Related to Figure 1; Supplementary Figure S2: Related to Figure 5; Supplementary Figure S3: (A) (top) Mouse TP53BP1 protein sequence marked with acetylated lysines (K1357, K1664) and functional segments. (B) Representative images of CTRL, Hdac1 KO SC, SAHA and MS-275 samples irradiated with 2 Gy ionizing irradiation and stained with DAPI (blue) and antibodies against γH2A.X (red) and TP53BP1 (green). (C) Representative images of CTRL, Hdac1 KO SC, SAHA and MS-275 samples stained with DAPI (blue) and antibodies against γH2A.X (red) and TP53BP1(green); Supplementary Table S1: Abundances of identified histone peptide forms in CTRL, SAHA, MS-275 and Hdac1 KO samples; Supplementary Table S2: Abundances of non-enriched proteins from non-chromatin fraction in SAHA, MS-275 and Hdac1 KO SC compared to CTRL; Supplementary Table S3: Abundances of identified acetylated peptides including those showing qualitative changes in SAHA, MS-275 and *Hdac1* KO SC compared to CTRL; Supplementary Table S4: List of mitochondrial proteins with downregulated acetylation following the HDACi treatments or *Hdac1* deletion, Supplementary Methods.

## References

[1] Verdin E., Ott M. (2015). 50 years of protein acetylation: From gene regulation to epigenetics, metabolism and beyond. Nat. Rev. Mol. Cell Biol..

[2] Haberland M., Montgomery R.L., Olson E.N. (2009). The many roles of histone deacetylases in development and physiology: Implications for disease and therapy. Nat. Rev. Genet..

[3] Narita T., Weinert B.T., Choudhary C. (2019). Functions and mechanisms of non-histone protein acetylation. Nat. Rev. Mol. Cell Biol..

[4] Stengel K.R., Hiebert S.W. (2015). Class I HDACs Affect DNA Replication, Repair, and Chromatin Structure: Implications for Cancer Therapy. Antioxid. Redox Signal.

[5] Lahue R.S., Frizzell A. (2012). Histone deacetylase complexes as caretakers of genome stability. Epigenetics.

[6] Roos W.P., Krumm A. (2016). The multifaceted influence of histone deacetylases on DNA damage signalling and DNA repair. Nucleic Acids Res..

[7] Faiola F., Liu X., Lo S., Pan S., Zhang K., Lymar E., Martinez E. (2005). Dual Regulation of c-Myc by p300 via Acetylation-Dependent Control of Myc Protein Turnover and Coactivation of Myc-Induced Transcription. Mol. Cell. Biol..

[8] Yuan Z., Guan Y., Chatterjee D., Chin Y.E. (2005). Stat3 Dimerization Regulated by Reversible Acetylation of a Single Lysine Residue. Science.

[9] Ikenoue T., Inoki K., Zhao B., Guan K.-L. (2008). PTEN Acetylation Modulates Its Interaction with PDZ Domain. Cancer Res..

[10] Tang Y., Zhao W., Chen Y., Zhao Y., Gu W. (2008). Acetylation Is Indispensable for p53 Activation. Cell.

[11] Guha M. (2015). HDAC inhibitors still need a home run, despite recent approval. Nat. Rev. Drug Discov..

[12] Jones P.A., Issa J.-P.J., Baylin S. (2016). Targeting the cancer epigenome for therapy. Nat. Rev. Genet..

[13] Suraweera A., O’Byrne K.J., Richard D.J. (2018). Combination Therapy with Histone Deacetylase Inhibitors (HDACi) for the Treatment of Cancer: Achieving the Full Therapeutic Potential of HDACi. Front. Oncol..

[14] Johnstone R.W. (2002). Histone-deacetylase inhibitors: Novel drugs for the treatment of cancer. Nat. Rev. Drug Discov..

[15] Xu W.S., Parmigiani R.B., Marks P.A. (2007). Histone deacetylase inhibitors: Molecular mechanisms of action. Oncogene.

[16] Balasubramanian S., Verner E., Buggy J.J. (2009). Isoform-specific histone deacetylase inhibitors: The next step?. Cancer Lett..

[17] Li W., Sun Z. (2019). Mechanism of Action for HDAC Inhibitors—Insights from Omics Approaches. Int. J. Mol. Sci..

[18] Dovey O.M., Foster C.T., Conte N., Edwards S.A., Edwards J.M., Singh R., Cowley S.M. (2013). Histone deacetylase 1 and 2 are essential for normal T-cell development and genomic stability in mice. Blood.

[19] Heideman M.R., Wilting R.H., Yanover E., Velds A., de Jong J., Kerkhoven R.M., Dannenberg J.H. (2013). Dosage-dependent tumor suppression by histone deacetylases 1 and 2 through regulation of c-Myc collaborating genes and p53 function. Blood.

[20] Santoro F., Botrugno O.A., Dal Zuffo R., Pallavicini I., Matthews G.M., Cluse L., Minucci S. (2013). A dual role for Hdac1: Oncosuppressor in tumorigenesis, oncogene in tumor maintenance. Blood.

[21] Grausenburger R., Bilic I., Boucheron N., Zupkovitz G., El-Housseiny L., Tschismarov R., Ellmeier W. (2010). Conditional Deletion of Histone Deacetylase 1 in T Cells Leads to Enhanced Airway Inflammation and Increased Th2 Cytokine Production. J. Immunol..

[22] Boucheron N., Tschismarov R., Goeschl L., Moser M.A., Lagger S., Sakaguchi S., Ellmeier W. (2014). CD4+ T cell lineage integrity is controlled by the histone deacetylases HDAC1 and HDAC. Nat. Immunol..

[23] Tschismarov R., Firner S., Gil-Cruz C., Göschl L., Boucheron N., Steiner G., Ellmeier W. (2014). HDAC1 Controls CD8+ T Cell Homeostasis and Antiviral Response. PLoS ONE.

[24] Morris S.W., Kirstein M.N., Valentine M.B., Dittmer K.G., Shapiro D.N., Saltman D.L., Look A.T. (1994). Fusion of a Kinase Gene, ALK, to a Nucleolar Protein Gene, NPM, in Non-Hodgkin’s Lymphoma. Science.

[25] Werner M.T., Zhang Q., Wasik M.A. (2017). From Pathology to Precision Medicine in Anaplastic Large Cell Lymphoma Expressing Anaplastic Lymphoma Kinase (ALK+ ALCL). Cancers.

[26] Chiarle R., Voena C., Ambrogio C., Piva R., Inghirami G. (2008). The anaplastic lymphoma kinase in the pathogenesis of cancer. Nat. Rev. Cancer..

[27] Amin H.M., Lai R. (2007). Pathobiology of ALK+ anaplastic large-cell lymphoma. Blood.

[28] Prokoph N., Larose H., Lim M.S., Burke G.A.A., Turner S.D. (2018). Treatment Options for Paediatric Anaplastic Large Cell Lymphoma (ALCL): Current Standard and beyond. Cancers.

[29] Kuchaříková H., Dobrovolná P., Lochmanová G., Zdráhal Z. (2021). Trimethylacetic Anhydride–Based Derivatization Facilitates Quantification of Histone Marks at the MS1 Level. Mol. Cell Proteom..

[30] Chiarle R., Gong J.Z., Guasparri I., Pesci A., Cai J., Liu J., Inghirami G. (2003). NPM-ALK transgenic mice spontaneously develop T-cell lymphomas and plasma cell tumors. Blood.

[31] Yamaguchi T., Cubizolles F., Zhang Y., Reichert N., Kohler H., Seiser C., Matthias P. (2010). Histone deacetylases 1 and 2 act in concert to promote the G1-to-S progression. Genes Dev..

[32] Činčárová L., Lochmanová G., Nováková K., Šultesová P., Konečná H., Fajkusová L., Zdráhal Z. (2012). A combined approach for the study of histone deacetylase inhibitors. Mol. Biosyst..

[33] Lochmanová G., Ihnatová I., Kuchaříková H., Brabencová S., Zachová D., Fajkus J., Fojtová M. (2019). Different Modes of Action of Genetic and Chemical Downregulation of Histone Deacetylases with Respect to Plant Development and Histone Modifications. Int. J. Mol. Sci..

[34] Perez-Riverol Y., Csordas A., Bai J., Bernal-Llinares M., Hewapathirana S., Kundu D.J., Vizcaíno J.A. (2019). The PRIDE database and related tools and resources in 2019: Improving support for quantification data. Nucleic Acids Res..

[35] Khan N., Jeffers M., Kumar S., Hackett C., Boldog F., Khramtsov N., Sehested M. (2007). Determination of the class and isoform selectivity of small-molecule histone deacetylase inhibitors. Biochem. J..

[36] Wang Z., Zang C., Cui K., Schones D.E., Barski A., Peng W., Zhao K. (2009). Genome-wide Mapping of HATs and HDACs Reveals Distinct Functions in Active and Inactive Genes. Cell.

[37] Mrakovcic M., Kleinheinz J., Fröhlich L.F. (2019). p53 at the Crossroads between Different Types of HDAC Inhibitor-Mediated Cancer Cell Death. Int. J. Mol. Sci..

[38] Winter M., Moser M.A., Meunier D., Fischer C., Machat G., Mattes K., Seiser C. (2013). Divergent roles of HDAC1 and HDAC2 in the regulation of epidermal development and tumorigenesis. EMBO J..

[39] Szklarczyk D., Gable A.L., Nastou K.C., Lyon D., Kirsch R., Pyysalo S., von Mering C. (2021). The STRING database in 2021: Customizable protein–protein networks, and functional characterization of user-uploaded gene/measurement sets. Nucleic Acids Res..

[40] Martínez-Reyes I., Chandel N.S. (2020). Mitochondrial TCA cycle metabolites control physiology and disease. Nat. Commun..

[41] Wardell S.E., Ilkayeva O.R., Wieman H.L., Frigo D.E., Rathmell J.C., Newgard C.B., McDonnell D.P. (2009). Glucose Metabolism as a Target of Histone Deacetylase Inhibitors. Mol. Endocrinol..

[42] Hoffmann M.J., Meneceur S., Hommel K., Schulz W.A., Niegisch G. (2021). Downregulation of Cell Cycle and Checkpoint Genes by Class I HDAC Inhibitors Limits Synergism with G2/M Checkpoint Inhibitor MK-1775 in Bladder Cancer Cells. Genes.

[43] Dong Z., Yang Y., Liu S., Lu J., Huang B., Zhang Y. (2017). HDAC inhibitor PAC-320 induces G2/M cell cycle arrest and apoptosis in human prostate cancer. Oncotarget.

[44] Luchenko V.L., Litman T., Chakraborty A.R., Heffner A., Devor C., Wilkerson J., Bates S.E. (2014). Histone deacetylase inhibitor-mediated cell death is distinct from its global effect on chromatin. Mol. Oncol..

[45] Nalawansha D.A., Gomes I.D., Wambua M.K., Pflum M.K.H. (2017). HDAC Inhibitor-Induced Mitotic Arrest Is Mediated by Eg5/KIF11 Acetylation. Cell Chem. Biol..

[46] Song M.S., Salmena L., Pandolfi P.P. (2012). The functions and regulation of the PTEN tumour suppressor. Nat. Rev. Mol. Cell Biol..

[47] Vega F., Medeiros L.J., Leventaki V., Atwell C., Cho-Vega J.H., Tian L., Rassidakis G.Z. (2006). Activation of Mammalian Target of Rapamycin Signaling Pathway Contributes to Tumor Cell Survival in Anaplastic Lymphoma Kinase–Positive Anaplastic Large Cell Lymphoma. Cancer Res..

[48] Turner S.D., Yeung D., Hadfield K., Cook S.J., Alexander D.R. (2007). The NPM-ALK tyrosine kinase mimics TCR signalling pathways, inducing NFAT and AP-1 by RAS-dependent mechanisms. Cell Signal.

[49] Robert C., Rassool F.V., Grant S. (2012). Chapter Three-HDAC Inhibitors: Roles of DNA Damage and Repair. Advances in Cancer Research.

[50] Choudhary C., Weinert B.T., Nishida Y., Verdin E., Mann M. (2014). The growing landscape of lysine acetylation links metabolism and cell signalling. Nat. Rev. Mol. Cell Biol..

[51] Weinert B.T., Iesmantavicius V., Moustafa T., Schölz C., Wagner S.A., Magnes C., Choudhary C. (2014). Acetylation dynamics and stoichiometry in Saccharomyces cerevisiae. Mol. Syst. Biol..

[52] Lee J.-H., Choy M.L., Ngo L., Foster S.S., Marks P.A. (2010). Histone deacetylase inhibitor induces DNA damage, which normal but not transformed cells can repair. Proc. Natl. Acad. Sci. USA.

[53] Lauffer B.E., Mintzer R., Fong R., Mukund S., Tam C., Zilberleyb I., Steiner P. (2013). Histone Deacetylase (HDAC) Inhibitor Kinetic Rate Constants Correlate with Cellular Histone Acetylation but Not Transcription and Cell Viability. J. Biol. Chem..

[54] Becher I., Dittmann A., Savitski M.M., Hopf C., Drewes G., Bantscheff M. (2014). Chemoproteomics reveals time-dependent binding of histone deacetylase inhibitors to endogenous repressor complexes. ACS Chem. Biol..

[55] Gong F., Miller K.M. (2013). Mammalian DNA repair: HATs and HDACs make their mark through histone acetylation. Mutat Res. Mol. Mech Mutagen..

[56] Sun Y., Jiang X., Chen S., Fernandes N., Price B.D. (2005). A role for the Tip60 histone acetyltransferase in the acetylation and activation of ATM. Proc. Natl. Acad. Sci. USA.

[57] Yuan Z., Seto E. (2007). A Functional Link Between SIRT1 Deacetylase and NBS1 in DNA Damage Response. Cell Cycle.

[58] Eliezer Y., Argaman L., Rhie A., Doherty A.J., Goldberg M. (2009). The Direct Interaction between 53BP1 and MDC1 Is Required for the Recruitment of 53BP1 to Sites of Damage. J. Biol. Chem..

[59] Nelson G., Buhmann M., von Zglinicki T. (2009). DNA damage foci in mitosis are devoid of 53BP. Cell Cycle.

[60] Belotserkovskaya R., Jackson S.P. (2014). Keeping 53BP1 out of focus in mitosis. Cell Res..

[61] Kelly R.D.W., Cowley S.M. (2013). The physiological roles of histone deacetylase (HDAC) 1 and 2: Complex co-stars with multiple leading parts. Biochem. Soc. Trans..

[62] Wagner G.R., Hirschey M.D. (2014). Nonenzymatic Protein Acylation as a Carbon Stress Regulated by Sirtuin Deacylases. Mol. Cell..

[63] Guo X., Bai Y., Zhao M., Zhou M., Shen Q., Yun C.H., Wang J. (2018). Acetylation of 53BP1 dictates the DNA double strand break repair pathway. Nucleic Acids Res..

[64] Kruglov O., Wu X., Hwang S.T., Akilov O.E. (2020). The synergistic proapoptotic effect of PARP-1 and HDAC inhibition in cutaneous T-cell lymphoma is mediated via Blimp-1. Blood Adv..

[65] Robert C., Nagaria P.K., Pawar N., Adewuyi A., Gojo I., Meyers D.J., Rassool F.V. (2016). Histone deacetylase inhibitors decrease NHEJ both by acetylation of repair factors and trapping of PARP1 at DNA double-strand breaks in chromatin. Leuk Res..

[66] Valdez B.C., Li Y., Murray D., Liu Y., Nieto Y., Champlin R.E., Andersson B.S. (2017). Combination of a hypomethylating agent and inhibitors of PARP and HDAC traps PARP1 and DNMT1 to chromatin, acetylates DNA repair proteins, down-regulates NuRD and induces apoptosis in human leukemia and lymphoma cells. Oncotarget.

