# Supplementary material for: Quantitative Acetylomics Uncover Acetylation-Mediated Pathway Changes Following Histone Deacetylase Inhibition in Anaplastic Large Cell Lymphoma
Source: Cells. 2022 Aug 2;11(15):2380. doi: 10.3390/cells11152380 (PMC9368142; doi:10.3390/cells11152380)
Supplement: Supplementary file 1 [file cells-11-02380-s001.zip › Supplementary Figures_merged.pdf]

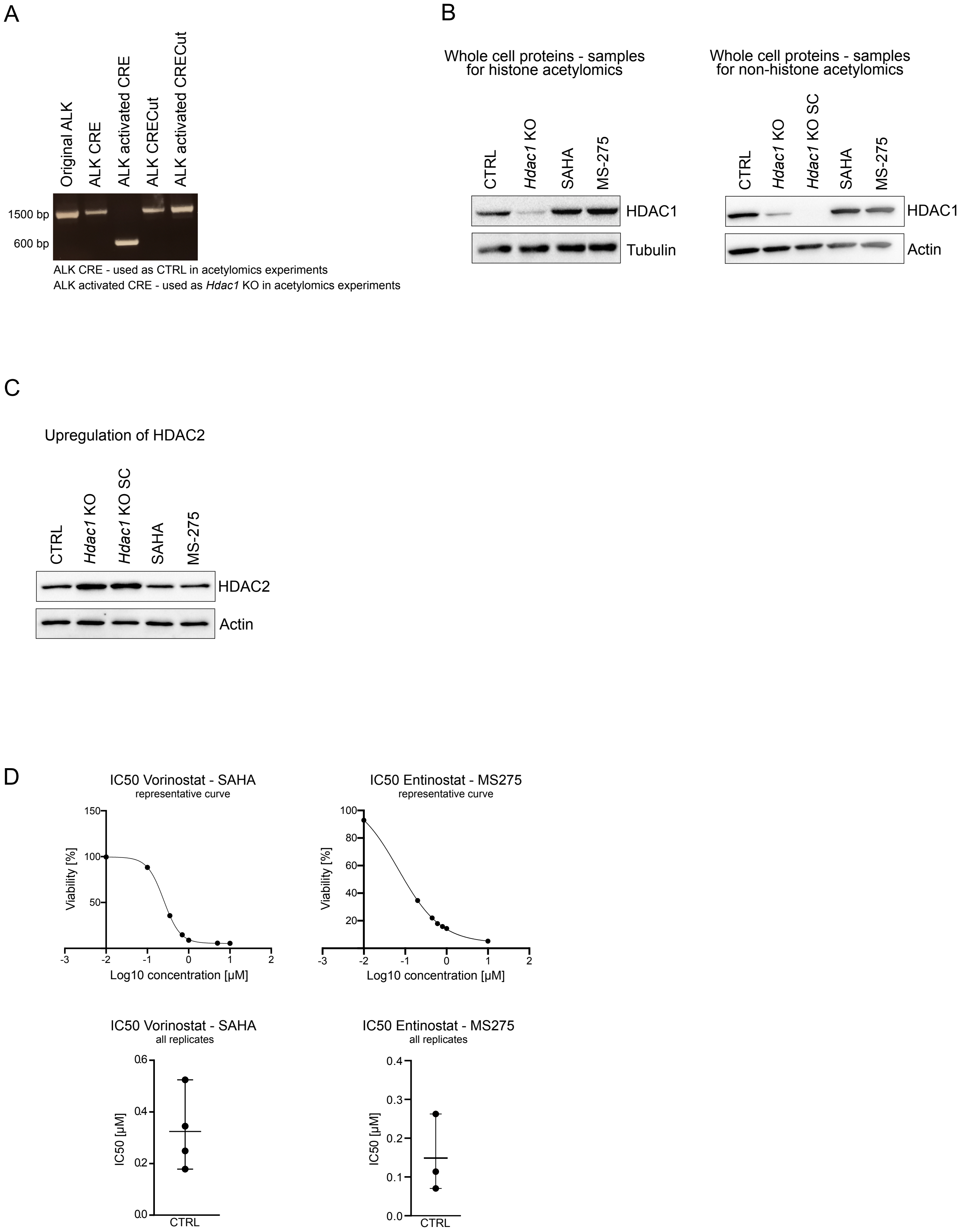
A
Original ALK
ALK CRE
ALK activated CRE
ALK CRECut
ALK activated CRECut
1500 bp
600 bp
ALK CRE - used as CTRL in acetylomics experiments
ALK activated CRE - used as *Hdac1* KO in acetylomics experiments
B
Whole cell proteins - samples for histone acetylomics
CTRL
Hdac1 KO
SAHA
MS-275
HDAC1
Tubulin
Whole cell proteins - samples for non-histone acetylomics
CTRL
Hdac1 KO
Hdac1 KO SC
SAHA
MS-275
HDAC1
Actin
C
Upregulation of HDAC2
CTRL
Hdac1 KO
Hdac1 KO SC
SAHA
MS-275
HDAC2
Actin
D
IC50 Vorinostat - SAHA
representative curve
Viability [%]
Log10 concentration [μM]
IC50 Entinostat - MS275
representative curve
Viability [%]
Log10 concentration [μM]
IC50 Vorinostat - SAHA
all replicates
IC50 [μM]
CTRL
IC50 Entinostat - MS275
all replicates
IC50 [μM]
CTRL

Supplementary Figure 1. ***Related to Figure 1.*** (**A**) Genotyping results for an ALK cell line isolated from a thymic tumor of an NPM-ALK *Hdac1*$^{fl/fl}$ transgenic mouse (original ALK), ALK cell line transduced with inducible CRE (ALK CRE), ALK cell line with inducible CRE where CRE was activated via tamoxifen treatment (ALK activated CRE), ALK cell line transduced with inducible CRE that was cut (ALK CRECut), ALK cell line with inducible CRE that was cut, where CRE was activated (ALK activated CRECut). For all further experiments "ALK CRE" was used as CTRL and "ALK activated CRE" was used as *Hdac1* KO. The bands at 1500 bp and 600 bp correspond to the floxed *Hdac1* gene and the delta *Hdac1*, respectively. (**B**) Confirmation of *Hdac1* knockout in *Hdac1* KO and *Hdac1* KO SC cell lines by Western blot analysis for following acetylomic analyses of histone and non-histone fractions. (**C**) Confirmation of HDAC2 upregulation upon *Hdac1* deletion by Western blot analysis. (**D**) (top) Representative IC50 curves for Vorinostat (SAHA) and Entinostat (MS-275), IC50 concentrations were determined after 48 h of treatment using viability as a read-out (as measured by resazurin). (bottom) Average of IC50 concentrations determined from multiple independent experiments.

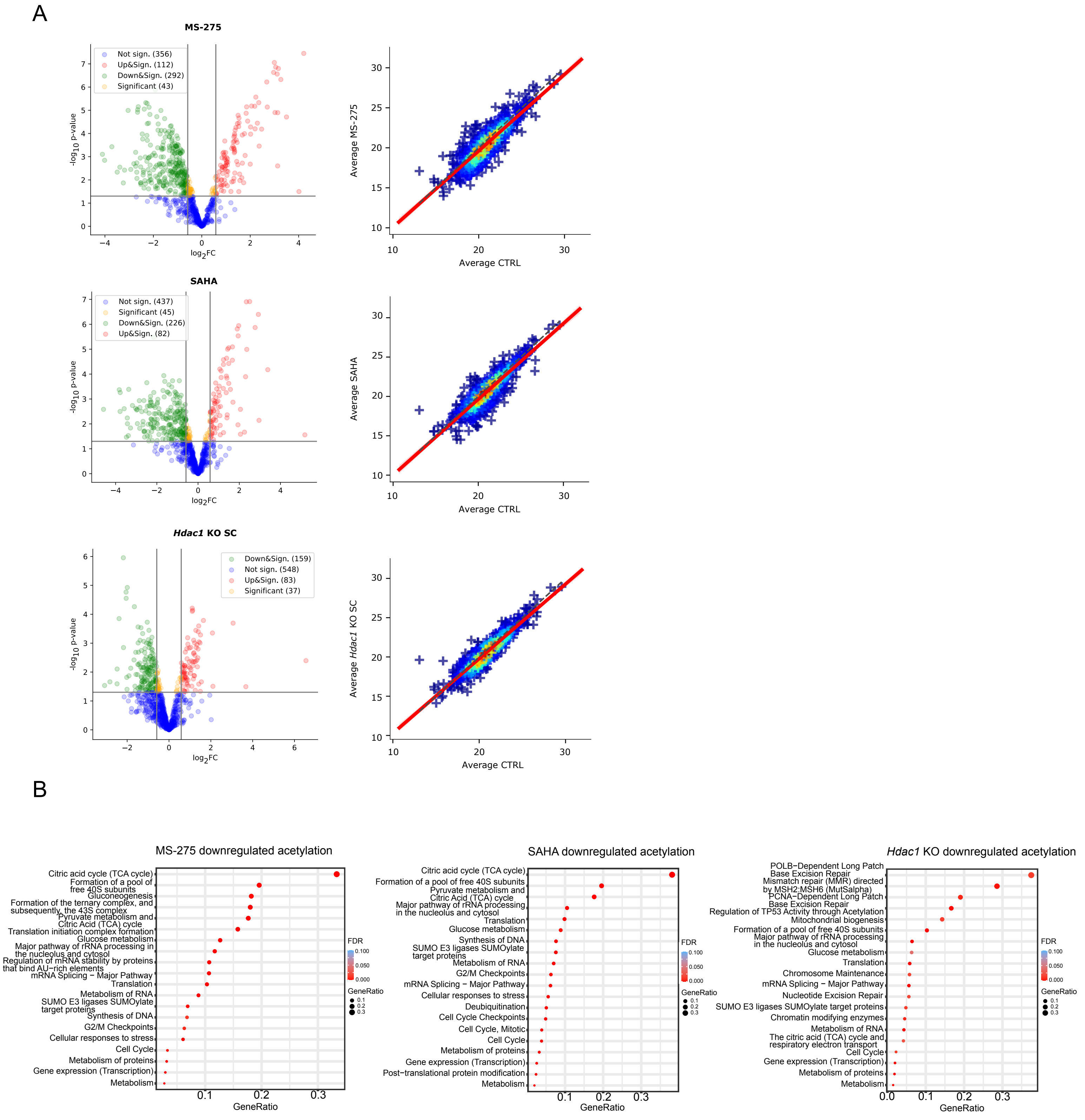
A
MS-275
Not sign. (356)
Up&Sign. (112)
Down&Sign. (292)
Significant (43)
-log10 p-value
log2FC
Average MS-275
Average CTRL
SAHA
Not sign. (437)
Significant (45)
Down&Sign. (226)
Up&Sign. (82)
Average SAHA
Hdac1 KO SC
Down&Sign. (159)
Not sign. (548)
Up&Sign. (83)
Significant (37)
Average Hdac1 KO SC
B
MS-275 downregulated acetylation
Citric acid cycle (TCA cycle)
Formation of a pool of free 40S subunits
Gluconeogenesis
Formation of the ternary complex, and subsequently, the 43S complex
Pyruvate metabolism and Citric Acid (TCA) cycle
Translation initiation complex formation
Glucose metabolism
Major pathway of rRNA processing in the nucleolus and cytosol
Regulation of mRNA stability by proteins that bind AU−rich elements
mRNA Splicing − Major Pathway
Translation
Metabolism of RNA
SUMO E3 ligases SUMOylate target proteins
Synthesis of DNA
G2/M Checkpoints
Cellular responses to stress
Cell Cycle
Metabolism of proteins
Gene expression (Transcription)
Metabolism
GeneRatio
FDR
GeneRatio
SAHA downregulated acetylation
Citric acid cycle (TCA cycle)
Formation of a pool of free 40S subunits
Pyruvate metabolism and Citric Acid (TCA) cycle
Major pathway of rRNA processing in the nucleolus and cytosol
Translation
Glucose metabolism
Synthesis of DNA
SUMO E3 ligases SUMOylate target proteins
Metabolism of RNA
G2/M Checkpoints
mRNA Splicing − Major Pathway
Cellular responses to stress
Deubiquitination
Cell Cycle Checkpoints
Cell Cycle, Mitotic
Cell Cycle
Metabolism of proteins
Gene expression (Transcription)
Post−translational protein modification
Metabolism
Hdac1 KO downregulated acetylation
POLB−Dependent Long Patch Base Excision Repair
Mismatch repair (MMR) directed by MSH2:MSH6 (MutSalpha)
PCNA−Dependent Long Patch Base Excision Repair
Regulation of TP53 Activity through Acetylation
Mitochondrial biogenesis
Formation of a pool of free 40S subunits
Major pathway of rRNA processing in the nucleolus and cytosol
Glucose metabolism
Translation
Chromosome Maintenance
mRNA Splicing − Major Pathway
Nucleotide Excision Repair
SUMO E3 ligases SUMOylate target proteins
Chromatin modifying enzymes
Metabolism of RNA
The citric acid (TCA) cycle and respiratory electron transport
Cell Cycle
Gene expression (Transcription)
Metabolism of proteins
Metabolism

Supplementary Figure 2. ***Related to Figure 5.*** (**A**) Illustration of changes in the levels of acetylated peptides between CTRL and samples with HDAC inhibition. In volcano plots, acetylated peptides with significantly upregulated levels are indicated in red (p-value < 0.05 and FC > 1.5), significantly downregulated in green (p-value < 0.05 and FC < 0.67), significant in yellow (p-value < 0.05 and 0.67 < FC < 1.5), and non-significant in blue (p-value > 0.05 and 0.67 < FC < 1.5). Log2-transformed abundances of acetylated peptides in CTRL and treated samples are compared in Scatter plots (**B**) REACTOME pathway analysis of proteins with downregulated acetylation levels in MS-275, SAHA and *Hdac1* KO SC samples compared to control (LIMMA test, p-value < 0.05, FC > 1.5), using the STRING network analysis tool.

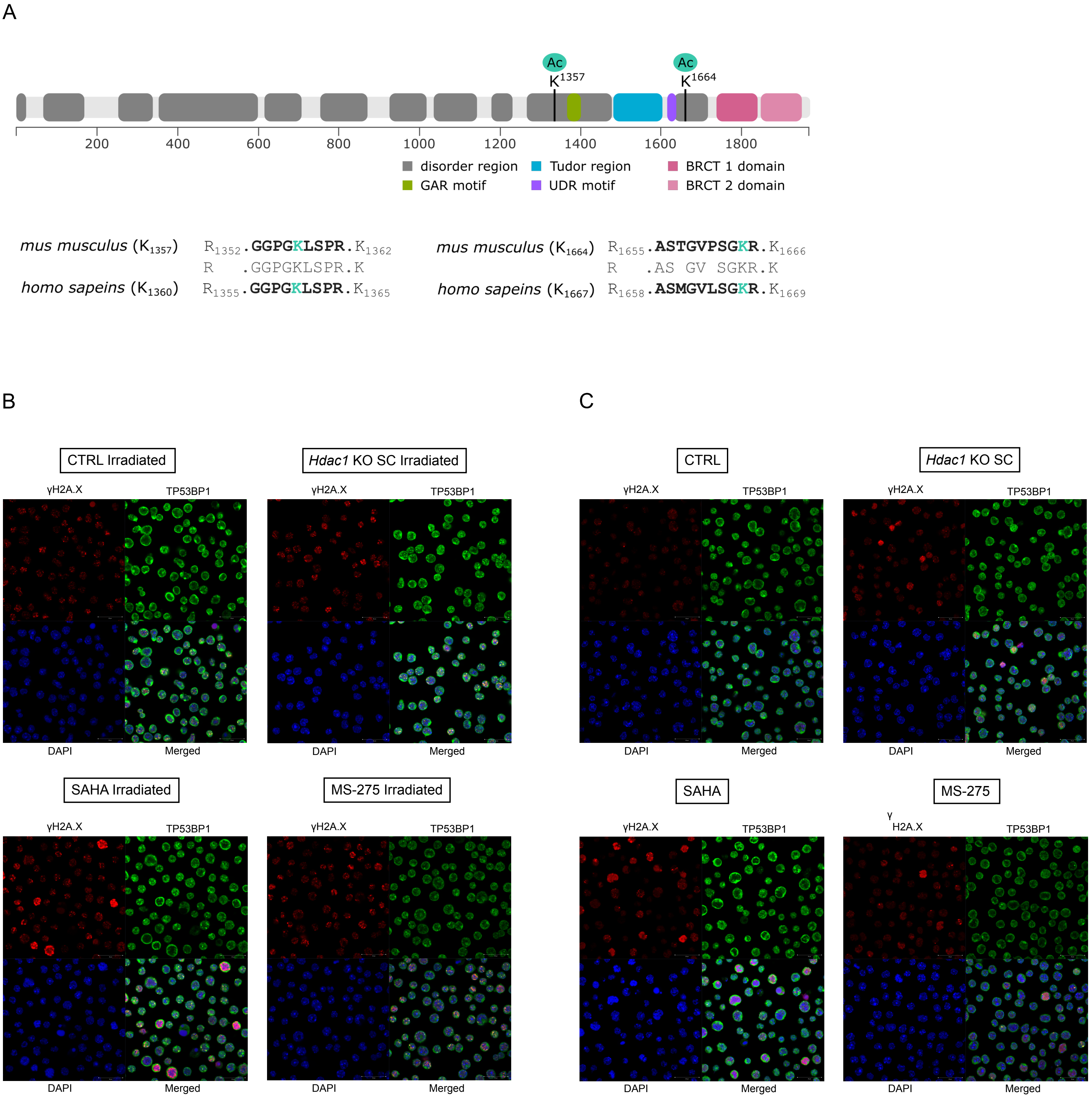
A
Ac
K1357
Ac
K1664
200
400
600
800
1000
1200
1400
1600
1800
disorder region
Tudor region
BRCT 1 domain
GAR motif
UDR motif
BRCT 2 domain
mus musculus (K1357)
R1352.GGPGKLSPR.K1362
R .GGPGKLSPR.K
homo sapeins (K1360)
R1355.GGPGKLSPR.K1365
mus musculus (K1664)
R1655.ASTGVPSGKR.K1666
R .AS GV SGKR.K
homo sapeins (K1667)
R1658.ASMGVLSGKR.K1669
B
C
CTRL Irradiated
Hdac1 KO SC Irradiated
CTRL
Hdac1 KO SC
γH2A.X
TP53BP1
DAPI
Merged
SAHA Irradiated
MS-275 Irradiated
SAHA
MS-275

Supplementary Figure 3. (**A**) (top) Mouse TP53BP1 protein sequence marked with acetylated lysines (K1357, K1664) and functional segments. Functional segments identified based on similarity with human protein (UniProt, MobiDB). (bottom) Alignment of acetylated peptides in mouse with their human protein analogues, depicting conserved lysine residues (K) in turquoise (**B**) Representative images of CTRL, *Hdac1* KO SC, SAHA and MS-275 samples irradiated with 2 Gy ionizing irradiation and stained with DAPI (blue) and antibodies against γH2A.X (red) and TP53BP1 (green). (**C**) Representative images of CTRL, *Hdac1* KO SC, SAHA and MS-275 samples stained with DAPI (blue) and antibodies against γH2A.X (red) and TP53BP1(green).
