# Supplementary material for: Quantitative Acetylomics Uncover Acetylation-Mediated Pathway Changes Following Histone Deacetylase Inhibition in Anaplastic Large Cell Lymphoma
Source: Cells. 2022 Aug 2;11(15):2380. doi: 10.3390/cells11152380 (PMC9368142; doi:10.3390/cells11152380)
Supplement: Supplementary file 1 [file cells-11-02380-s001.zip › Supplementary Methods.pdf]

Histone extract preparation

Histone extracts were prepared according to published protocol (1). Briefly, cells were washed twice with ice cold PBS, incubated in lysis buffer consisting of 80 mM NaCl, 20 mM EDTA (Sigma, E5134), 1% Triton X-100 (Millipore, 648466), 45 mM sodium butyrate (Aldrich, 303410) and 0.1 mM PMSF (Sigma, P7626) and frozen at -80 °C for several days. After thawing, samples were centrifuged at 2000 g for 8 min. Each pellet was resuspended in 500 µL of ice-cold H2SO4 (Penta, Czech Republic) and incubated at 4 °C for 2 h. Supernatant was cleared by centrifugation at 16000 g and 4 °C for 8 min, then histones were precipitated during 30 min incubation with 500 µL of 50% ice cold trichloroacetic at 0 °C with shaking. The precipitate was harvested by centrifugation at 5000 g at 4 °C for 30 min, washed with 50 mM HCl (Penta) in acetone, twice with acetone and dried at vacuum concentrator. The prepared histone extract was dissolved in water and Bradford Assay (Bio-Rad, CA, USA) was used for protein concentration measurement.

Histone derivatization

Histone derivatization was performed according to previously published procedure (2). Briefly, 12 µg of histone extract were diluted to a final concentration of 1 µg.µL 1 with 50% (v/v) acetonitrile (ACN; Honeywell, NC, USA). The pH was adjusted to 8 with NH4OH and 3 µL of derivatization reagent consisting of trimethylacetic anhydride (Sigma-Aldrich, MO, USA) and ACN in a 1:3 (v/v) ratio was added. The sample was incubated for 5 h at RT with shaking, followed by repeated derivatization step including 16 h incubation. Subsequently, sample proceeded two rounds of microwave-assisted histone derivatization, as follows. Sample volume was reduced to 5 µL in vacuum concentrator and 50% (v/v) ACN was added to a final volume of 12 µL. Each round included three derivatization sub-cycles consisting of sample's pH adjustment to 8 with NH4OH, addition of 3 µL of derivatization reagent, and two 1 min incubation in the microwave oven at 350 W (short spin between incubations). Microtube with sample was covered with glass beaker during incubation in microwave oven. After two complete rounds (6 additions of reagent in total) sample volume was reduced to 5 µl, and 0.3 µg of trypsin (Sequencing grade modified, Promega Corporation, WI, USA) in 40 µL of 100 mM ammonium bicarbonate (ABC) was added and sample was incubated at 37 °C for 4 h followed by another addition of 0.3 µg of trypsin for 12 h incubation. Digested sample underwent two rounds of above-described microwave-assisted derivatization for labelling of newly released peptide N-termini. After first round, sample was diluted to a final volume of 24 µL, and completely dried out after the second round.

Preparation of non-chromatin peptides for acetylomic analysis

About 1×108 cells per sample were harvested and washed twice in ice cold PBS. Each cell pellet was resuspended in 10 ml of urea lysis buffer consisting of 9 M urea in 20 mM HEPES, pH 8.0 with 45 mM sodium butyrate, and 5 M NaCl was added to a final concentration of 50 mM. Precipitated chromatin was separated from non-chromatin cell fraction by centrifugation (4000 g for 10 min). Supernatant was carefully transferred to a new tube and frozen at -80 °C.

Protein concentration in thawed sample was determined by Micro BCA™ Protein Assay Kit (Thermo Fisher Scientific, MA, USA). Following steps were performed according to PTMScan® Acetyl-Lysine Motif [Ac-K] Kit (Cell Signaling Technology, MA, USA) manufacturer instructions with minor changes. Proteins' disulfide bonds were reduced by 30 min incubation with 4.5 mM dithiothreitol at 55 °C. Sample was briefly cooled to RT on ice and cysteine residues were alkylated by 15 min incubation with 10 mM iodoacetamide in dark. Sample was 3-times diluted with 20 mM HEPES and SOLu Trypsin at E:S ratio 1:100 (w/w) was added for overnight protein digestion at RT. The digest was acidified with trifluoroacetic acid (TFA) to pH < 3 and purified on SepPak C18 cartridge (360mg sorbent per cartridge, Waters, MA, USA). First, cartridge sorbent was reconditioned by 5 ml of 100% ACN followed by sequential equilibration with 1 ml, 3 ml, and 6 ml of 0.1% TFA. Then sample was loaded to the cartridge and sequentially washed with 1 ml, 5 ml, and 6 ml of 0.1% TFA. Cleared peptides were eluted in three steps, each with 2 ml of 0.15% TFA in 40% ACN, frozen at -80 °C, and lyophilized for 2 days to remove TFA.
Purified non-chromatin peptides were reconstituted in 1.4 ml of PTMScan IAP buffer (Cell Signaling Technology), cleared by centrifugation at 10000 g at 4 °C for 5 min and peptide concentration was determined by Micro BCA™ Protein Assay Kit. An aliquot of each sample was taken to prepare non-enriched peptide mixture at concentration of 1 μg.μL 1 in 1% formic acid (FA, Merck Millipore, MA, USA) for LC-MS/MS analysis.
PTMScan Acetyl-Lysine Motif beads (Cell Signaling Technology) were used for acetylated peptides affinity enrichment. Before incubation, beads were 4 times pre-washed with 1 ml of PBS by inverting tube few times in hands and cleared by centrifugation at 2000 g at 4 °C for 30 s. One aliquot of beads was incubated with 3 mg of peptides for each sample at 4 °C for 2 h with mild rotation. Immunoprecipitate was gently washed twice with 1 ml of PBS and three times with 1 ml of HPLC-grade water. Enriched peptides were eluted in two steps, each with 55 μL of 0.15% TFA.

Purification of samples prior LC-MS/MS analysis

Prior LC-MS/MS analysis, both histone and non-chromatin peptide samples were desalted on HyperSep SpinTip C18 (Thermo Fisher Scientific) according to manufacturer recommendations. Derivatized histones were diluted with 0.1% TFA before desalting, and sequentially eluted with 0.1% TFA in 50% ACN and 0.1% TFA in 75% ACN. Enriched acetylated peptides were eluted sequentially with 0.1% TFA in 40% ACN. All samples were dried in vacuum concentrator to remove TFA and reconstituted in 0.1% FA (Honeywell) before LC MS/MS analysis. Non-enriched non-chromatin peptides were additionally purified by phase-separation with water-saturated ethyl acetate according to published protocol (3), dried in vacuum concentrator and reconstituted in 0.1% FA (Honeywell).

If not specified, all chemicals were purchased from Sigma – Aldrich, MO, USA.

LC-MS/MS analysis

The samples of all peptide mixtures – 1) derivatized histone peptides, 2) non-chromatin peptides, and 3) enriched acetylated peptides of non-chromatin cell fraction – were analysed

on RSLCnano liquid chromatograph connected to an Orbitrap Fusion Lumos TriBrid mass spectrometer (Thermo Fisher Scientific). Analytical column outlet was directly connected to a Digital PicoView 550 ion source with PicoTip SilicaTip (New Objective, MA, USA) or Captive spray (ZDV, Bruker, MA, USA) emitter. Active Background Ion Reduction Device (ESI Source Solutions, MA, USA) was installed.

Derivatized histones were analysed according to published protocol (2). In outline, peptides were concentrated on trap column packed with X Bridge BEH 130 C18 sorbent (100 µm x 30 mm, 3.5 µm particles, Waters). Chromatographic separation was performed on Acclaim Pepmap100 C18 analytical column (75 µm x 500 mm, 3 µm particles, Thermo Fisher Scientific) with mixture of mobile phases A (0.1% FA in water) and B (0.1% FA in 80% ACN). Both columns were equilibrated and tempered at 40 °C before sample injection. Peptides were eluted within 85 min gradient at 300 nL.min-1 flow rate and increasing content of mobile phase B: from 5 to 25 % in 20 min, from 25 to 29 % in 10 min, from 29 to 32 % in 10 min, from 32 to 38 % in 15 min, from 38 to 50 % in 20 min, and from 50 to 85 % in 10 min. Separation was finished with 10 min isocratic wash with 85% B.

Both peptide mixtures of non-chromatin cell fraction were analysed under the same chromatographic conditions. First, peptides were concentrated and desalted on Pepmap100 C18 µPrecolumn trap column (300 µm x 5 mm, 5 µm particles, Thermo Fisher Scientific) tempered at 40 °C and then separated on Acclaim Pepmap100 C18 analytical column (75 µm x 500 mm, 3 µm particles, Thermo Fisher Scientific) at ambient temperature (25 °C). The equilibration of both columns was done prior sample injection. Peptides were eluted within 110 min gradient at increasing content of mobile phase B: from 1 % to 35 % in 100 min, then from 35 % to 80 % in 10 min at flow rate of 300 nL.min-1.

Mass spectrometry data were acquired in data-dependent mode. Survey scan parameters were mass range 350 – 2000 m/z, resolution 60 000 (at m/z 200), and target value 4×105 for all peptide mixtures. Cycle time between master scans was 3 s for histones and non-enriched peptides, and 2 s for enriched peptides. Maximum injection time for master scan was 54 ms, 50 ms and 500 ms for histone, non- enriched and enriched peptides, respectively. Precursors with charge state between 2+ to 7+ exceeding intensity 1×104 (5×104 for non-enriched peptides) were selected in Quadrupole (isolation window 1.6 m/z). Tandem mass spectra of HCD fragmented precursors (30% normalized collision energy) were acquired at 30 000 Orbitrap resolution (at m/z 200). Ions for MS/MS analysis were accumulated until they reached intensity of 5×104, but no longer than 500 ms (54 ms for non-enriched peptides). After one MS/MS spectra acquisition, precursors were excluded for 30 s (60 s for histone peptides) before the next fragmentation.

Data analysis

All acquired raw data were searched against the cRAP universal contamination database (based on http://www.thegpm.org/crap/, 112 sequences), an in-house Mus Musculus histone database (v210309, 106 protein sequences, generated from UniProt) and UniProt KB Mouse database (v201007, taxon ID:10090, 21989 sequences) using an in house Mascot search engine (v2.6.2, Matrix Science, United Kingdom) through Proteome Discoverer software (v2.2.0.388, Thermo Fisher Scientific).

Derivatized histone peptides' search settings were described in previous work (2) with minor change. The mass error tolerance for precursors was 7 ppm (10 ppm for cRAP database), and 0.03 Da (0.5 Da for cRAP database) for fragments. Variable modifications selected for cRAP database search were Acetylation (K), Deamidation (N, Q), Oxidation (M), and Trimethylacetylation (N-term, K, S, T, Y). Histone database variable modifications were the same as for UniProt KB Mouse database – Acetylation (K, Protein N-term) and Trimethylacetylation (N-term, K, S, T, Y). Additional variable modifications for histone database were Methylation (K, R), Dimethylation (K), Trimethylation (K) and Phosphorylation (S, T). For all databases semiArg-C enzyme specificity with 2 allowed missed cleavages was set. Results were refined by fixed value PSM validator (delta Cn <0.05). Identifications of selected histone peptides were manually inspected, their quantity was determined with Skyline software (v19.1. or higher) based on peak areas in EICs.
Search parameters for data of non-chromatin cell fraction were as follows. Trypsin enzyme specificity with 3, 5, and 8 allowed missed cleavages was set for cRAP, UniProt KB, and Histone database, respectively. For all databases, Carbamidomethylation (C) was set as a static modification. The mass error tolerances were 7 ppm and 0.3 Da (0.02 Da for Histone database) for precursors and fragments, respectively. Variable modifications in cRAP database were Acetylation (K, Protein N-term) and Oxidation (M). The same variable modifications were selected for Histone and UniProt KB database – Acetylation (K, Protein N-term), Methylation (K, R), Dimethylation (K), and Trimethylation (K).
The search results of non-chromatin peptides were refined by applying 1 % FDR q-value based validation in Percolator node. Label-free quantification was carried out in Proteome discoverer software with combination of Minora Feature Detector, Feature Mapper and Precursor Ion Quantifier nodes with default settings. Only peptides with high confidence, minimal 6 amino acid length, and Mascot Ion Score at least 30 were used for further processing.

Statistics

The relative abundances of histone peptides were evaluated according to previously published methodology (4) using R script in KNIME Analytics Platform. Briefly, peptides' peak areas were treated as compositions, missing values were imputed, and areas were transformed to relative abundances. The quantitative data from replicates were combined by geometric mean and closure and multiply transformed, t-tests were used to compute the differences between sample groups and peptide forms (presented in log2 ratios of relative abundances).
Peptide level data and protein list were used for statistical evaluation of non-chromatin acetyl-lysine enriched and non-enriched samples, respectively. From protein list only proteins with two or more peptides were considered. From both data sets cRAP hits were excluded. Peptide and protein abundance data were log2 transformed, then subjected to median normalization. Only peptides and proteins identified in more than one sample per sample group proceeded two-group comparison using the LIMMA statistical test for differential expression with Benjamin-Hochberg p-value adjustment. Fold change (FC) >1.5 with p-value <0.05 was considered as significant for both peptide and protein results. Results with significant change in protein quantity were excluded. Peptide results were connected to corresponding protein data. The KNIME Analytics platform was used for statistical evaluation of data.

References

1. Činčárová L, Lochmanová G, Nováková K, Šultesová P, Konečná H, Fajkusová L, et al. A combined approach for the study of histone deacetylase inhibitors. Mol Biosyst. 2012 Oct 2;8(11):2937–45.

2. Kuchaříková H, Dobrovolná P, Lochmanová G, Zdráhal Z. Trimethylacetic Anhydride–Based Derivatization Facilitates Quantification of Histone Marks at the MS1 Level. Mol Cell Proteomics. 2021 Jan 1;20:100114.

3. Yeung Y-G, Stanley ER. Rapid Detergent Removal from Peptide Samples with Ethyl Acetate for Mass Spectrometry Analysis. Curr Protoc Protein Sci. 2010;59(1):16.12.1-16.12.5.

4. Lochmanová G, Ihnatová I, Kuchaříková H, Brabencová S, Zachová D, Fajkus J, et al. Different Modes of Action of Genetic and Chemical Downregulation of Histone Deacetylases with Respect to Plant Development and Histone Modifications. Int J Mol Sci. 2019 Jan;20(20):5093.
